# A physical reservoir involving frequency virtual nodes for structural damage detection

**DOI:** 10.1038/s41598-026-56840-8

**Published:** 2026-06-26

**Authors:** Konosuke Takashima, Naoko Watanabe, Motoaki Hiraga, Nanako Miura, Arata Masuda

**Affiliations:** 1 Division of Mechanophysics, Kyoto Institute of Technology, Kyoto, 606-8585 Japan; 2 Faculty of Mechanical Engineering, Kyoto Institute of Technology, Kyoto, 606-8585 Japan

**Keywords:** Structural health monitoring, Physical reservoir computing, Damage classification, Virtual node, Classification, Neural network, Civil engineering, Mechanical engineering

## Abstract

This study proposes a physical reservoir computing approach for structural damage detection, in which the target structure itself serves as the physical reservoir. Physical reservoir computing is a computational framework that leverages the dynamics of physical systems as computational resources for processing time series data. The proposed physical reservoir in this study is a nonlinear feedback system, which involves frequency virtual nodes realized by dividing the measured response of the target structure into multiple frequency bands. The divided signals are then respectively passed through nonlinear activation functions, followed by being summed and fed back to the target structure. The structure with the frequency virtual nodes and nonlinear feedback acts as a high-dimensional physical reservoir even with a single pair of sensor and actuator, which enables damage classification by detecting the changes in its dynamical behavior. To validate the proposed approach, both numerical and experimental verifications on a thin plate and a thin-walled tube with a single pair of sensor and actuator were conducted. As a result, the proposed method achieved comparable damage classification accuracy to conventional neural networks, while significantly reducing the training and inference costs, demonstrating the potential of a novel framework of structural health monitoring based on the physical reservoir computing.

## Introduction

To ensure the sustainable prosperity and safety of society, there is a growing demand for the efficient, highly automated maintenance of infrastructures and industrial facilities. Such technologies have been extensively researched in the field of structural health monitoring (SHM) over the past two decades. Despite the early technologies rather based on physical modeling^1,2^, the majority of the SHM technologies have been shifting to data-driven approaches^3^, involving the collection of time series data, such as vibration data from a large number of sensors, and utilizing high-performance computational resources to analyze this data for damage assessment and decision-making regarding structural integrity.

In the field of data-driven SHM, the application of artificial intelligence and machine learning technologies is actively studied and implemented. Technologies such as support vector machines^4–6^, deep neural networks (DNN)^7–9^ and convolutional neural networks^10^ are commonly used, which enable the damage detection by extracting features from time series data. Additionally, recurrent neural network models such as long short-term memory (LSTM)^11^ and gated recurrent units^12^ take raw time series data as input to detect damages. These applications are increasingly deployed on cloud platforms^13^, which provide high-performance computing environments with fast processing speeds and abundant memory resources. However, several challenges have emerged as the complexity and scale of the neural networks hosted on the cloud have rapidly grown^14^. These challenges include the saturation of data traffic due to the concentration of vast amounts of sensor data on the cloud, increased power consumption and communication volume associated with large-scale neural networks, extended training time, and issues related to communication latency. To address these challenges, edge computing^15^ is a promising solution to mitigate the saturation of data traffic due to data centralization and the computational burden of large-scale processing. This is done by locally performing computations on devices at the edge of the network, enabling to make the volume of the data transmitted to the cloud significantly reduced.

**Fig. 1:**
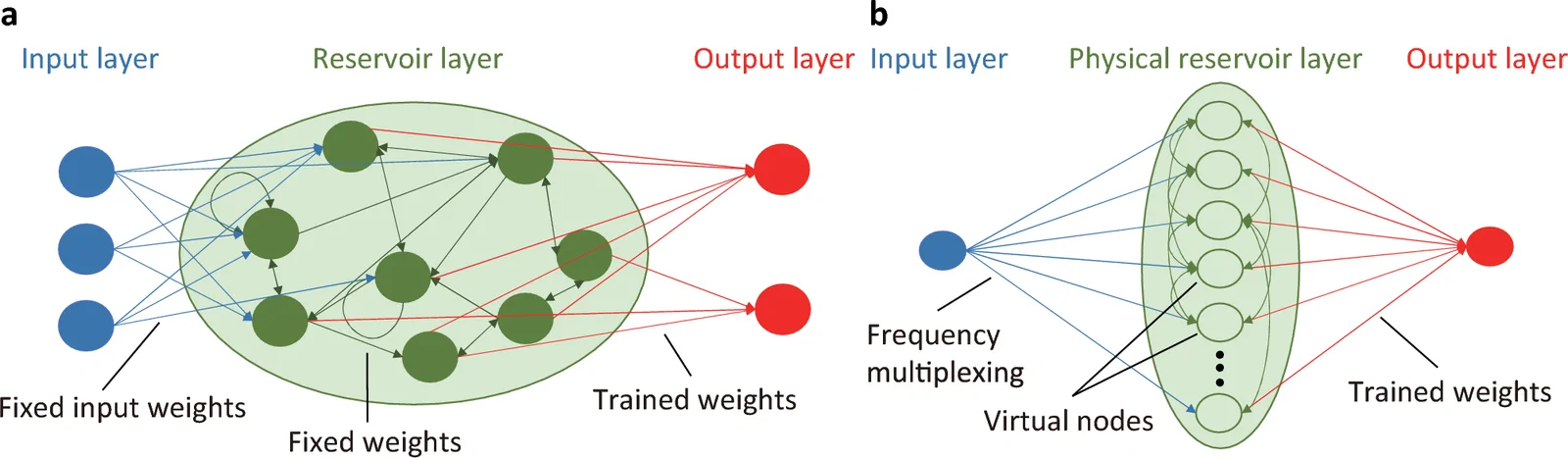
Reservoir computing schemes. (**a**) Classical reservoir computing scheme. The input layer consists of one or more input nodes, which are connected to the reservoir layer containing *N* nodes with randomly determined weights. The *N* nodes within the reservoir layer are interconnected with randomly determined weights. The weights connecting the reservoir layer to the output layer are adjusted through linear learning. (**b**) Frequency virtual node-based physical reservoir (FVN-PR) scheme. A single input node excites the physical system. The weights from the input layer to the physical reservoir layer are defined by the frequency response functions of the physical system and band-pass filters used for frequency division. The *N* virtual nodes within the physical reservoir layer are interconnected by nonlinear functions and bandwidth overlap (Fig. 2(b)). The weights connecting the physical reservoir layer to the output layer are adjusted through linear learning.

**Fig. 2:**
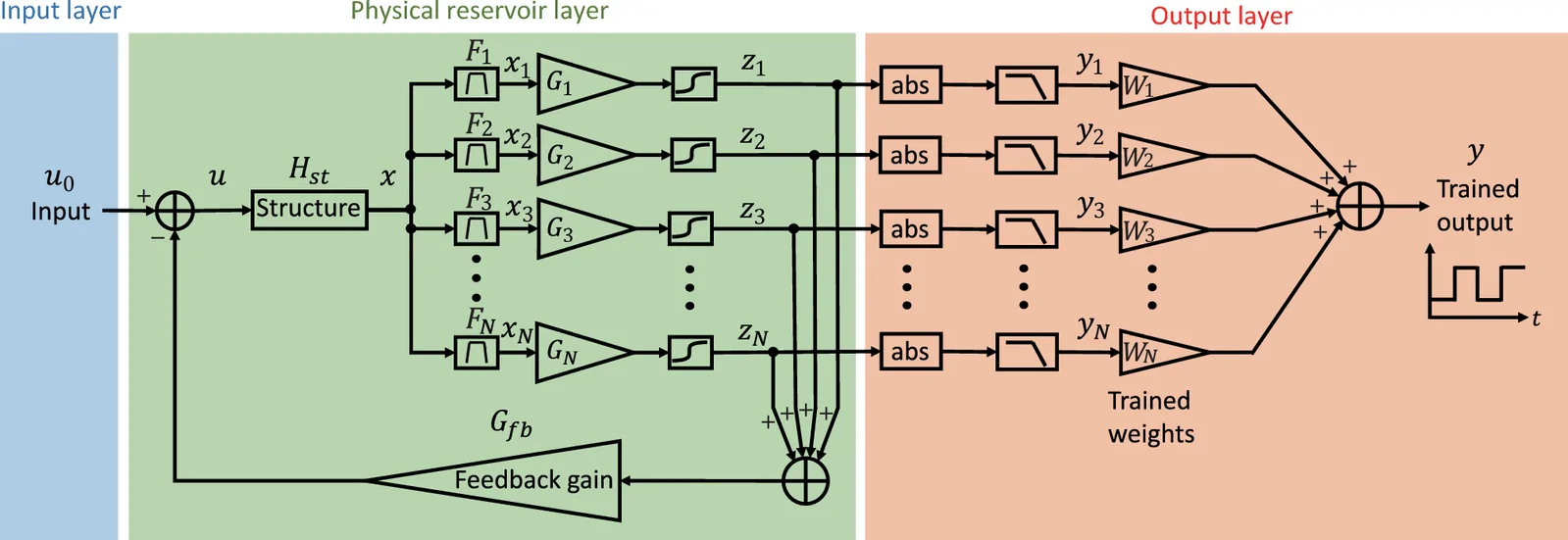
Design of connections of virtual nodes. (**a**) Connection topology of virtual nodes. The blue lines represent local connections, while the red lines represent global connections. (**b**) Frequency response functions of some virtual nodes. Each virtual node is a third-order Butterworth band-pass filter with a bandwidth of $f_\textrm{w} = 20$ Hz. Each virtual node is designed to have a center frequency ranging from 30 to 1020 Hz, with a center frequency spacing of *Δ f = 10* Hz between the adjacent virtual nodes.

Currently, physical reservoir computing (PRC) is gaining attention as a potential method for processing information at the edge^16^. PRC is a computational framework that leverages physical dynamics as computational resources. Examples of PRC include a reservoir composed of nonlinear mass-spring systems^17^, octopus-like robotic limbs^18^, spintronic PRC^19–22^, and optical reservoir computing^23–25^. Reservoir computing (RC)^26,27^, the predecessor of PRC, is a computational framework that employs recurrent neural networks (RNN) for pattern recognition in time series and sequential data. Early works include echo state networks^28^ and liquid state machines^29^. A typical RC system consists of three parts: input layer, reservoir layer, and output layer (Fig. 1(a)). The input data are simply delivered through the input layer to the reservoir layer, a mutually connected neural network, and read out through the output layer. Unlike the conventional RNNs, only the output weights are to be learned by linear regression, while the other weights are initially determined randomly and remain fixed. This simplicity makes it possible to replace the reservoir layer with a specific physical system, leading to the concept of PRC.

The key features required for the physical reservoir (PR) are the same as those for the reservoir, i.e., nonlinearity, high-dimensionality, and short-term memory^30–32^. Nonlinearity and high-dimensionality serve the same role as the kernel method^33^, mapping data to a high-dimensional feature space. Short-term memory is essential for ensuring the asymptotic stability of the system. When these three requirements are appropriately fulfilled, PRC systems achieve rich expressive power, enabling tasks such as spoken digit recognition^34^, signal classification^35^, pattern recognition^36^, and prediction^37^ with low computational costs.

Aligning with the context of PRC, Masuda et al.^38,39^ proposed a concept of SHM that utilizes the structure itself as a computational resource to detect its own damages. In this concept, the target structure was treated as a PR in the same way as in the conventional PRC, but the main focus was on detecting changes in the PR itself due to structural damages, rather than performing some computational tasks on the input as the conventional PRC did. The major challenge in this concept is how to turn the target structure into a PR, which should satisfy the three requirements mentioned above. Among those, the easiest to meet is short-term memory, because the structure itself is stable, and all that should be kept in mind is to ensure that it does not become unstable due to some parasitic /exogenous feedback mechanism. On the other hand, nonlinearity is difficult to realize, because the structures subject to health monitoring are typically linear dynamical systems. The earliest attempt^39^ proposed to attach nonlinear mass-spring systems to the structure, but this was obviously impractical and inefficient. High-dimensionality is a problem of how to secure the number of independent output sequences drawn from the PR, which is typically thought to be 100 to 1000. A straightforward approach would be to install such number of sensors and data acquisition systems on the PR, but this is not only impractical, but also undermines the benefits of introducing PRC at all.

In this study, a method to be more realistic and effective is proposed, that is, a method that combines the concept of virtual nodes with nonlinear mapping and feedback mechanism using a small number of sensors and actuators. The concept of virtual nodes aims to achieve high-dimensionality by employing multiplexing techniques. This concept was first named by Appeltant et al.^40^, presenting delay-time reservoir computing using time-division multiplexing. Following their proposal, various innovations have emerged, particularly in the field of optical reservoir computing, including reservoir computing utilizing wavelength-division multiplexing^41,42^ and frequency-division multiplexing^43^.

In this paper, virtual nodes based on frequency division are adopted, as they are likely easier to implement than time-division virtual nodes (Fig. 1(b)). Intentional band overlap and nonlinear functions embedded in the feedback loop are introduced to ensure coarse connections between frequency virtual nodes, and the saturation characteristics of the nonlinear functions also serve to bound the growth of the node response within a finite range, which is caused by the instability intentionally introduced by the high-gain feedback. With these tricks, the three requirements for a reservoir are intended to be guaranteed. The configured PRC thus learns the dynamic characteristics of the PR itself, which inherently incorporates the target structure into the feedback loop; as a result, changes in the PR, that is, changes in the dynamic behavior of the target structure caused by damage, can be evaluated, such as through a classification problem. As a minimum configuration, this paper examines the validity of the proposed method in the case of a single sensor and a single actuator setup by numerical and experimental verification. In the numerical verification part, the detection of the structural damage is performed as a two-class classification problem with intact and damaged classes for simplicity assuming that the damaged cases are predetermined and the training data can be acquired in advance. In the experimental tests, multi-class classification and regression problems are also addressed.

## Design of frequency virtual node-based PR for structural damage detection

### Architecture

**Fig. 3:**
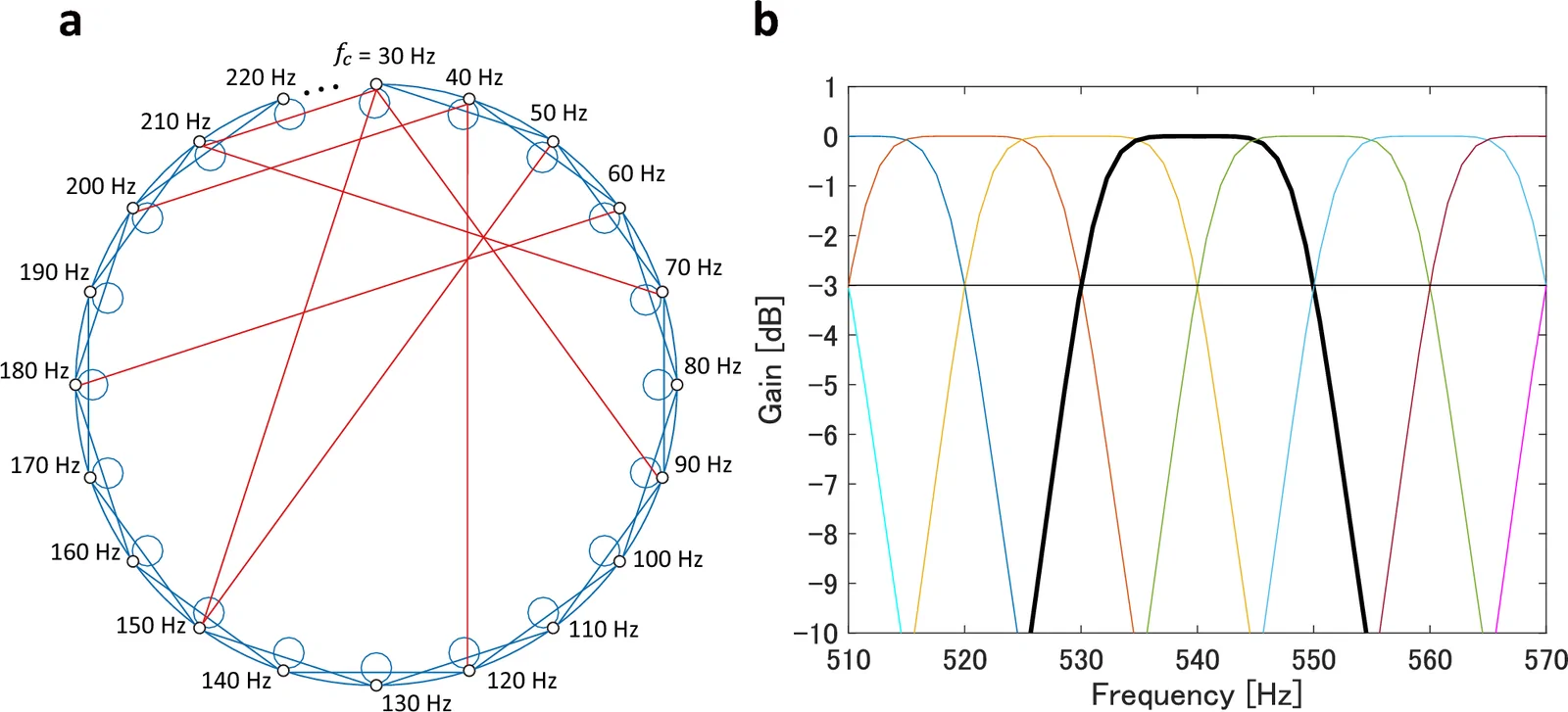
Frequency virtual node-based PR (FVN-PR) for SHM. The input is an external excitation signal, denoted by $u_0$, which is summed with the feedback signal to form the input *u*, and applied to the structure via a single actuator. The dynamical response of the structure is measured by a single sensor, denoted by *x*, which is then decomposed into multiple-band signals, $x_1, \ldots , x_N$ using a bank of band-pass filters $F_1(\omega ), \ldots , F_N(\omega )$ with an equal bandwidth but shifted center frequencies. Each band of the filter bank plays the role of the virtual node in the reservoir layer, and the number of the filters *N* corresponds to the number of the nodes. The divided signals are then respectively amplified by node gains $G_1, \ldots , G_N$, followed by being passed through *\tanh* activation functions to form virtual node signals $z_1, \ldots , z_N$. All virtual node signals are summed, multiplied by a feedback gain $G_\textrm{fb}$, and then added back to the input. In the output layer, the virtual node signals undergo absolute value computation, followed by low-pass filtering, and are finally linearly trained to approximate the desired output.

Figure 3 illustrates the architecture of the proposed frequency virtual node-based PR (FVN-PR) for structural damage detection. The input is an external excitation signal, denoted by $u_0$, which is summed with the feedback signal to form the input *u*, and applied to the structure via a single actuator. The dynamical response of the structure is measured by a single sensor, denoted by *x*, which is then decomposed into multiple-band signals, $x_1, \ldots , x_N$ using a bank of band-pass filters $F_1(\omega ), \ldots , F_N(\omega )$ with an equal bandwidth but shifted center frequencies. Each band of the filter bank plays the role of the virtual node in the reservoir layer, and the number of the filters *N* corresponds to the number of the nodes. The divided signals are then respectively amplified by node gains $G_1, \ldots , G_N$, followed by being passed through *\tanh* activation functions to form virtual node signals $z_1, \ldots , z_N$ . All virtual node signals are summed, multiplied by a feedback gain $G_\textrm{fb}$, and then added back to the input. These relationships are formulated as follows in the time domain:

1 $$\begin{aligned} u(t)=u_0(t)-G_\textrm{fb}\sum _{i=1}^{N} z_{i}(t) \end{aligned}$$

2 $$\begin{aligned} z_{i}(t) = \tanh (G_{i}x_{i}(t)) \end{aligned}$$

and in the frequency domain as

3 $$\begin{aligned} \tilde{x_{i}}(\omega ) = F_{i}(\omega )\tilde{x}(\omega ) \end{aligned}$$

4 $$\begin{aligned} \tilde{x}(\omega )=H_{\textrm{st}}(\omega )\tilde{u}(\omega ) \end{aligned}$$

where the variables with tilde represent the Fourier transform of the corresponding variables, and $H_{\textrm{st}}(\omega )$ is the frequency response function of the structure.

The input $u_{0}$ is to be chosen to effectively stimulate all virtual nodes. The node gain $G_{i}$ is introduced to normalize the magnitude of the node signal before it is passed through the activation function. It is determined node by node by first examining the root mean square value of $\hat{x}_i$, the band-pass filtered structural response excited only by the input without the feedback, and then use it to normalize the magnitude as follows:

5 $$\begin{aligned} G_{i} = \frac{\gamma }{\textrm{RMS}\{\hat{x}_i\}} \end{aligned}$$

where *γ* is a common gain and $\textrm{RMS}\{\cdot \}$ is the root mean square value. The above gain $G_{i}$ has the effect of approximately equalizing the magnitude of the node signal before the activation function to the level of *γ*.

The output layer is depicted in the right half of Fig. 3, in which the absolute value of the node signal $z_i$ is taken, followed by applying a low-pass filter, to extract the output $y_i$ for each virtual node, which quantifies the intensity of the node signal. The cut-off frequency of the low-pass filter is chosen equal to the virtual node bandwidth $f_\textrm{w}$. The output *y* is a linear sum of $y_1, \ldots , y_N$ with trainable weights $W_1, \ldots , W_N$, which are to be trained by linear learning with L2 regularization.

### Connection topology of virtual nodes

If there is no nonlinearity in the network and the virtual node bandwidth is independent of each other, there will be no connections between the virtual nodes, and the reservoir will not be sufficiently “rich”. To introduce appropriate connections between nodes, two techniques are adopted in the physical reservoir proposed in this study. One is bandwidth overlap, which introduces local node-to-node connections. By setting the half-power bandwidth of the band-pass filter bank to a value larger than the center frequency spacing, the bandwidth of adjacent nodes is overlapped. This overlap allows information from one node to propagate to adjacent nodes through the feedback loop. The other leverages the effect of the nonlinear element. By applying an odd-symmetric nonlinear activation function (tanh in this study), the virtual node signals are distorted, and odd-order harmonics are added to the narrow-band node signal. As consequence, information from a certain node affects nodes with a frequency band of its odd-numbered multiple through the feedback loop. This creates global connections across distant frequency bands, in contrast to the local connections provided by bandwidth overlap.

Figure 2(a) schematically shows the topology of these connections. The blue lines represent local connections, while the red lines represent global connections. These provide the connectivity required for the reservoir layer, and the hybrid structure of local and global connections contributes to ensuring the sparseness of node-to-node connections, which is an important factor in providing “rich” expressiveness to RC^28,31,32^.

## Numerical verification

### Two-class classification problem description

In order to demonstrate the potential ability of the proposed FVN-PR, we define the structural damage detection task as a two-class classification problem, based on the assumption that it is possible to obtain training data in advance for the case of intact and damaged structures. Needless to say, this assumption is unrealistic; for designing more practical damage detection tasks, we need to consider multi-class or single-class classification framework. Leaving these as future challenges, our primary objective here is to verify the expressive capability of the proposed PR in the simplest two-class classification problem.

**Fig. 4:**
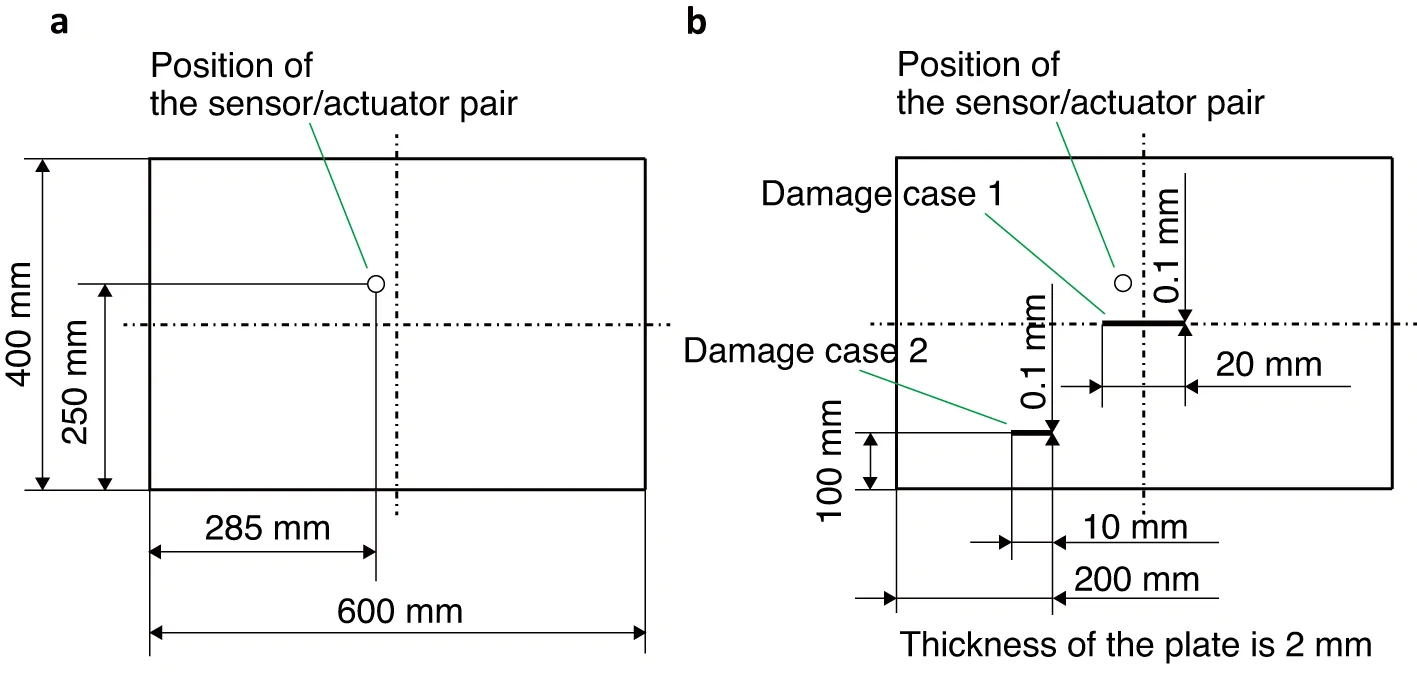
Illustrations of intact and damaged states of the target structure. (**a**) Intact state of the target structure. A clamped rectangular thin plate made of aluminum with a sensor/actuator pair installed at the location illustrated in the figure. (**b**) Damaged state of the target structure. Damage case 1 is defined as a state where a straight slit of 0.1 mm in width and 20 mm in length is created at the center of the plate. Damage case 2 is defined as a state where a straight slit of 0.1 mm in width and 10 mm in length is made at the location illustrated in the figure.

The target structure is assumed to be a clamped rectangular thin plate made of aluminum depicted in Fig. 4(a), and the data for the verification experiment is prepared using a numerical simulation. The proposed FVN-PR is implemented numerically for an intact plate and a damaged plate, a plate with a straight slit as a simulated crack. The location and size of the simulated crack is prepared in two cases as shown in Fig. 4(b). A co-located point displacement sensor and force actuator pair is assumed to be installed at the location depicted in Fig. 4(a).

The dynamical response of the plate is calculated by solving mode-decomposed equations of motion given by

6 $$\begin{aligned} \ddot{\xi }_{mn}(t) + 2\zeta _{mn} \omega _{mn} \dot{\xi }_{mn}(t) + \omega _{mn}^2 \xi _{mn}(t) = \Phi _{mn}u(t) \end{aligned}$$

where $\xi _{mn}$, $\zeta _{mn}$, $\omega _{mn}$, and $\Phi _{mn}$ denote the modal displacement, modal damping ratio, natural frequency, and mass-normalized eigenmode function at the sensor/actuator location of (*m*, *n*) mode, respectively; further, *u* is the point force from the actuator, which is defined by Eqs. (1), (2), and (3). The structural response measured by the point sensor is calculated as

7 $$\begin{aligned} x(t) = \sum _{m,n} \Phi _{mn}\xi _{mn}(t) \end{aligned}$$

Note that Eqs. (6) and (7) are the concrete description of the input-output relationship expressed by Eq. (4).

A white noise is used as the input signal $u_0$ in Eq. (1) so that all nodes could be evenly excited, and the PR output *y* is calculated by performing numerical response analysis. The desired output is set to 0 for intact cases and 1 for damaged cases, and the weights in the output layer are determined using linear learning with L2 regularization. The classification performance is then verified using the validation data generated using a different white noise series.

### Design of frequency virtual nodes

Figure 2(b) shows the frequency bands of the virtual nodes. The adjacent virtual nodes are intentionally designed to overlap, enabling interconnections between the virtual nodes through feedback. The number of virtual nodes is *N = 100*. Each virtual node uses a third-order Butterworth band-pass filter with a bandwidth of $f_\textrm{w} = 20$ Hz. The virtual nodes are designed to have a center frequency ranging from 30 to 1020 Hz, with a center frequency spacing of *Δ f = 10* Hz between the adjacent virtual nodes. The eigenmodes of the plate cover the range from (1, 1) mode to (8, 1) mode.

### Performance indexes

To evaluate the damage classification accuracy of the proposed method, two performance indexes are employed. The first is the area under the ROC curve (AUC). The receiver operating characteristic (ROC) curve is a visual tool used to assess the performance of a classifier by plotting the relationship between the True Positive Rate (TPR) and False Positive Rate (FPR). TPR represents the proportion of actual positive samples that are correctly classified, which is also known as Sensitivity or Recall. TPR is defined as follows:

8 $$\begin{aligned} \textrm{TPR} = \frac{\textrm{TP}}{\textrm{TP} + \textrm{FN}} \end{aligned}$$

where TP denotes the number of true positive samples, and FN denotes the number of false negative samples. Since this study involves time series data, positive and negative samples are counted for each time sample. Conversely, FPR represents the proportion of actual negative samples that are incorrectly classified as positive, which is also known as the fall-out. FPR is defined as follows:

9 $$\begin{aligned} \textrm{FPR} = \frac{\textrm{FP}}{\textrm{FP} + \textrm{TN}} \end{aligned}$$

where FP represents the number of false positives, and TN denotes the number of true negatives. The ROC curve is constructed by plotting TPR on the vertical axis and FPR on the horizontal axis. The area under the drawn ROC curve is called the AUC, which ranges from 0 to 1. A value closer to 1 indicates a higher performance of the classifier.

The second is the root mean square error (RMSE) between the desired output $y_d(t)$ and trained output *y*(*t*) for the validation signal. Given the number of time samples *S*, the performance index $E_\textrm{rms}$ is expressed by the following equation:

10 $$\begin{aligned} E_\textrm{rms} = \sqrt{\frac{1}{S} \sum _{t=1}^{S}|y_d(t)-y(t)|^2} \end{aligned}$$

### Classification performance

Damage classification tests were conducted using a data sequence prepared by simulating the PRC response to the white noise input by solving the equations described above with a sampling frequency of 10 kHz. In the data sequence, the first and the next 25-second parts are training data for the structure in the intact and damaged states, respectively. Meanwhile, the second half of the sequence starting from 50 second is served for the validation; the part from 50 second is for the intact structure while the part from 75 second is for the damaged structure. Each 25-second data sequence originally lasted 35 seconds, with the first 10 seconds removed to eliminate the effects of transient responses.

**Fig. 5:**
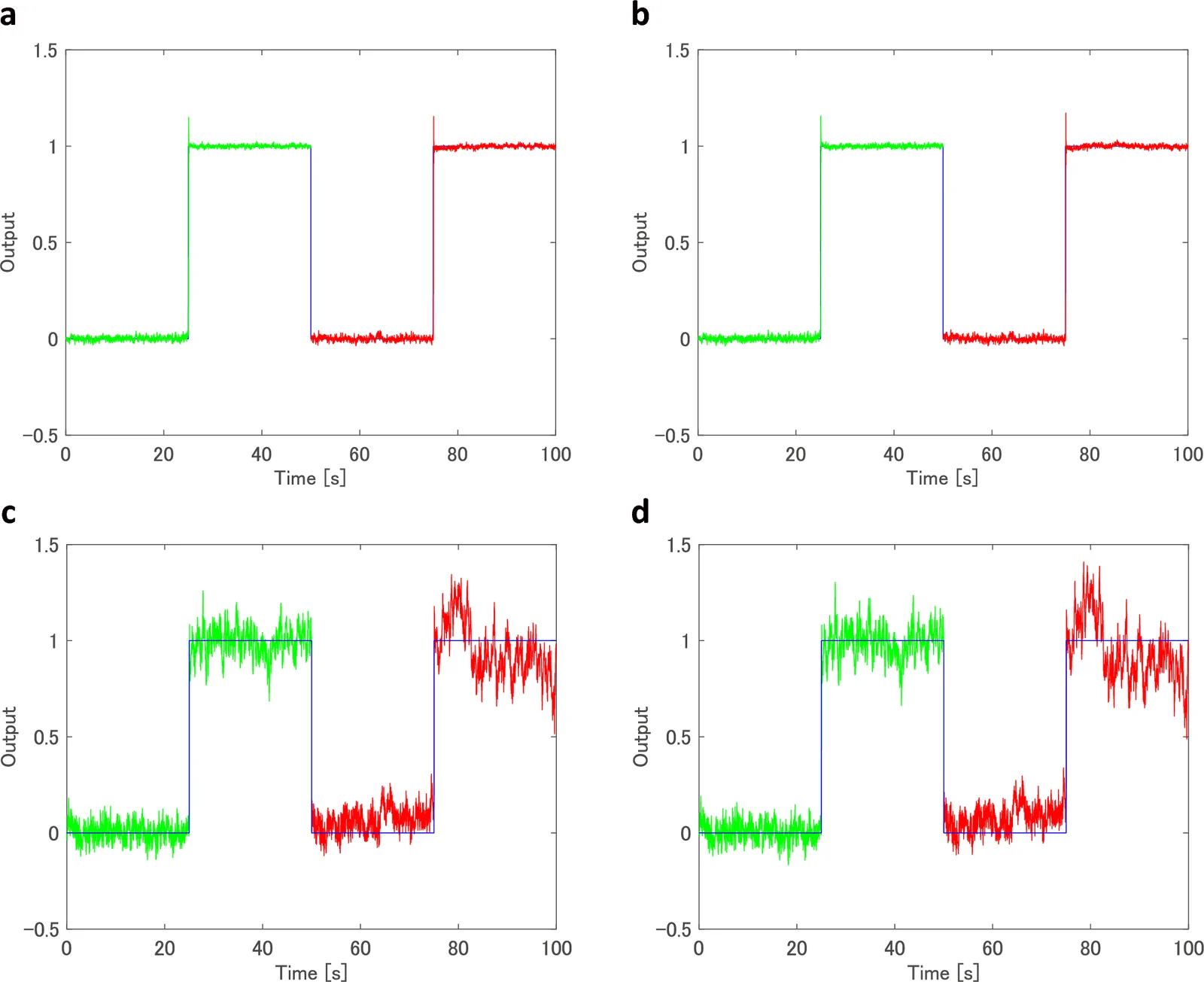
Results of damage classification using the proposed method, along with results showing the contribution of feedback and nonlinearity. The virtual node characteristics are the same as in Fig. 2 ($f_\textrm{w} = 20$ Hz, *Δ f = 10* Hz, *N = 100*). The parameters of the physical reservoir layer are set to a common gain of *γ = 0.1* and a feedback gain of $G_\textrm{fb} = 1$. The green and red lines represent the learning results for the training data and validation data, respectively, while the blue line represents the desired output $y_d(t)$. The desired output $y_d(t)$ is a time series signal where 0 indicates an intact state and 1 indicates a damaged state. (**a**) Results for damage case 1 using the proposed method with both the feedback and nonlinear function ($\textrm{AUC} = 0.99936$, $E_\textrm{rms} = 0.0313$). (**b**) Results for damage case 2 using the proposed method with both the feedback and nonlinear function ($\textrm{AUC} = 0.99927$, $E_\textrm{rms} = 0.0316$). (**c**) Results for damage case 1 without the feedback ($\textrm{AUC} = 0.99987$, $E_\textrm{rms} = 0.1274$). (**d**) Results for damage case 1 without both the feedback and nonlinear function ($\textrm{AUC} = 0.99992$, $E_\textrm{rms} = 0.1420$).

Figures 5 (a) and (b) show the results of damage classification using the proposed method. The virtual node characteristics are the same as in Fig. 2 ($f_\textrm{w} = 20$ Hz, *Δ f = 10* Hz, *N = 100*). The parameters of the physical reservoir layer are set to a common gain of *γ = 0.1* and a feedback gain of $G_\textrm{fb} = 1$. The green and red lines represent the learning results for the training data and validation data, respectively, while the blue line represents the desired output $y_d(t)$. As shown in Fig. 5(a) and (b), the output of the network was sufficiently close to the desired values achieving almost perfect classification except for the moments when the output changes from 0 to 1. The performance indexes $\textrm{AUC}$ and $E_\textrm{rms}$ for the damage case 1 were $\textrm{AUC} = 0.99936$ and $E_\textrm{rms} = 0.0313$, while those for the damage case 2 were $\textrm{AUC} = 0.99927$ and $E_\textrm{rms} = 0.0316$.

To evaluate how the nonlinearity and feedback affect classification, the results are shown for the case without the feedback (Fig. 5(c)) and the case without both the feedback and nonlinear function (Fig. 5(d)). Note that the results for the case without the nonlinear function are not presented, as the absence of the nonlinear saturation function caused the network to become unstable. As shown in Fig. 5(c) and (d), the outputs of the network were more randomly varied compared with the results in 5(a) and (b), and even showed a drifting tendency for the validation data. The values of $E_\textrm{rms}$ deteriorated significantly, to 0.1274 in the case without the feedback and 0.1420 in the case without both the feedback and nonlinear function. The corresponding $\textrm{AUC}$ values were 0.99987 and 0.99992, respectively, showing no significant change. This is because the above-mentioned variation in the output barely affected the discrimination with a threshold of 0.5. The fact that the results in Fig. 5(c) and (d) are almost identical indicates that the saturated nonlinear function is only meaningful when feedback is activated. This is understandable because the effect of the saturating nonlinear function, yielding the network stability and global inter-node coupling, only becomes apparent when the feedback is present.

The results above show that the nonlinear function and feedback in the proposed method are functioning effectively. In order to understand how these contribute to improve the damage classification performance, it is necessary to look into the internal response of the network in more detail. This will be further discussed in the next section.

**Fig. 6:**
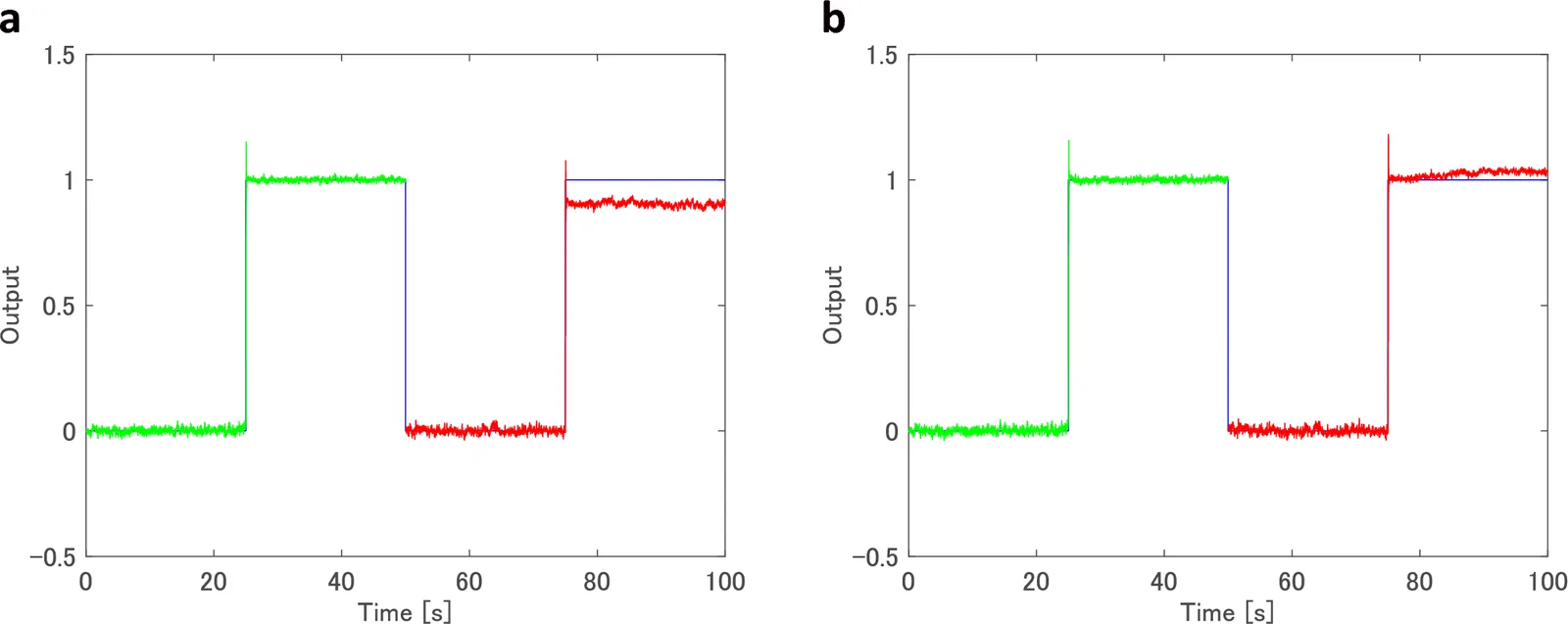
Results of damage classification when training data and validation data have different damage cases. The virtual node characteristics are the same as in Fig. 2 ($f_\textrm{w} = 20$ Hz, *Δ f = 10* Hz, *N = 100*). The parameters of the physical reservoir layer are set to a common gain of *γ = 0.1* and a feedback gain of $G_\textrm{fb} = 1$. The green and red lines represent the learning results for the training data and validation data, respectively, while the blue line represents the desired output $y_d(t)$. The desired output $y_d(t)$ is a time series signal where 0 indicates an intact state and 1 indicates a damaged state. (**a**) Results for the proposed method, where the damaged state in the training data corresponds to damage case 1 and the damaged state in the validation data corresponds to damage case 2 ($\textrm{AUC} = 0.99936$, $E_\textrm{rms} = 0.0750$). (**b**) Results for the proposed method, where the damaged state in the training data corresponds to damage case 2 and the damaged state in the validation data corresponds to damage case 1 ($\textrm{AUC} = 0.99928$, $E_\textrm{rms} = 0.0361$).

Next, to verify the generalization performance of the proposed method, the physical reservoir trained with the dataset for damage case 1 was validated using the dataset for damage case 2, and vice versa. The virtual nodes and the parameters of the physical reservoir in this test are the same as those in Fig. 5(a) and (b). The results plotted in Fig. 6(a) and (b) show that the classification was successful despite a slight deviation in the results for the validation data. For the performance indexes, the results for the proposed method when the training data corresponded to damage case 1 and the validation data to damage case 2 are $\textrm{AUC} = 0.99936$ and $E_\textrm{rms} = 0.0750$, and when the training data corresponded to damage case 2 and the validation data to damage case 1, the results are $\textrm{AUC} = 0.99928$ and $E_\textrm{rms} = 0.0361$. Although these results are merely a specific example, they suggest that the FVN-PR has a certain level of generalization performance.

### Parameter study

In this section, a parameter study was conducted to investigate the parameters influencing the classification performance of the FVN-PR, such as the common gain *γ*, feedback gain $G_{\textrm{fb}}$, number of virtual nodes *N*, and virtual node bandwidth $f_\textrm{w}$. The default values of the parameters were set as follows: common gain *γ = 0.1*, feedback gain $G_\textrm{fb} = 1$, virtual node bandwidth $f_\textrm{w} = 20$ Hz, center frequency spacing between adjacent virtual nodes *Δ f = 10* Hz, and number of virtual nodes *N = 100*. All the experiments were repeated 10 times, and the root mean square of the RMSE values was calculated.

**Fig. 7:**
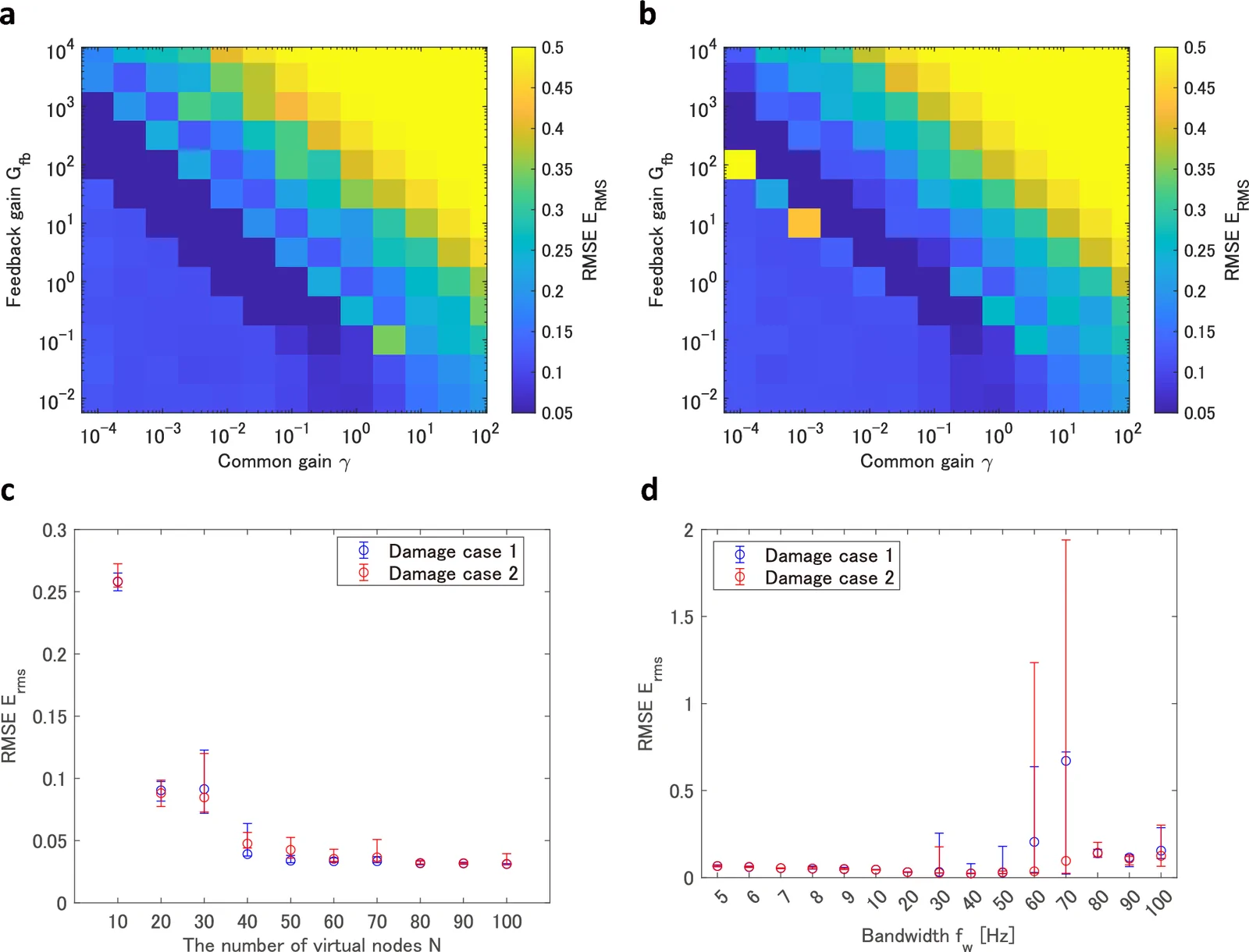
Parameter study results. All the experiments were repeated 10 times. (**a**) Results of the parameter study for the common gain *γ* and feedback gain $G_\textrm{fb}$ for the validation data of damage case 1. The color map shows the root mean square of the RMSE values. The virtual node characteristics are the same as in Fig. 2 ($f_\textrm{w} = 20$ Hz, *Δ f = 10* Hz, *N = 100*). (**b**) Results of the parameter study for the common gain *γ* and feedback gain $G_\textrm{fb}$ for the validation data of damage case 2. The color map shows the root mean square of the RMSE values. The virtual node characteristics are the same as in Fig. 2 ($f_\textrm{w} = 20$ Hz, *Δ f = 10* Hz, *N = 100*). (**c**) The RMSE values when the number of virtual nodes *N* was varied from 10 to 100. The circles and error bars indicate the median values and maximum/minimum values, respectively, and RMSEs for the validation data with damage case 1 (blue) and damage case 2 (red) are represented. The parameters of the physical reservoir layer are set to a common gain of *γ = 0.1* and feedback gain of $G_\textrm{fb} = 1$. The virtual node bandwidth is $f_\textrm{w} = 20$ Hz, and center frequency spacing between adjacent virtual nodes is *Δ f = 10* Hz. (**d**) The RMSE values are shown when the virtual node bandwidth $f_\textrm{w}$ was varied from 5 Hz to 100 Hz. The circles and error bars indicate the median values and maximum/minimum values, respectively, and RMSEs for the validation data with damage case 1 (blue) and damage case 2 (red) are represented. The parameters of the physical reservoir layer are set to a common gain of *γ = 0.1* and feedback gain of $G_\textrm{fb} = 1$. The number of virtual nodes is *N = 100*, and center frequency spacing between adjacent virtual nodes is *Δ f = 10* Hz.

Firstly, the roles of the common gain *γ* and feedback gain $G_{\textrm{fb}}$ were investigated. Figure 7(a) and (b) represent color maps of the RMSE for various combination of the common gain *γ* and feedback gain $G_{\textrm{fb}}$, for the damage case 1 and 2, respectively. All the other parameters were fixed as the default values. From the figures, one can observe an inverse proportional relationship between the common gain *γ* and feedback gain $G_{\textrm{fb}}$ giving good damage classification accuracy. This is understandable because the open-loop gain is constant along the diagonal direction. The best damage classification performance is exhibited along the line expressed as $\gamma \times G_{\textrm{fb}} = 0.1$, and the common gain *γ* is under 1. (the dark blue region in Fig. 7 (a) and (b)). When both the common gain *γ* and the feedback gain $G_{\textrm{fb}}$ are high (upper-right yellow region of the Fig. 7(a) and (b)), the RMSE reaches 0.5, indicating a complete failure in damage classification. Conversely, when both the common gain *γ* and the feedback gain $G_{\textrm{fb}}$ are low (lower-left light blue region of the Fig. 7(a) and (b)), neither nonlinearity nor feedback functions effectively, resulting in outcomes similar to those shown in Fig. 5 (d).

To understand how the gains affect the damage classification accuracy, the response of each virtual node was closely investigated. (See **Supplementary Information** online.) The analysis revealed that, since this network is a feedback system with the saturation element *\tanh*, the virtual node response *z* can exhibit either asymptotic stability or limit cycle behavior. When the gain is appropriately set, as indicated by the dark blue region in Fig. 7 (a) and (b), some of the virtual nodes exhibit limit cycle behavior with an amplitude exceeding 0.5 as one can observe in Supplementary Fig. S1 online, in which the absolute values of the temporal responses of all virtual nodes are plotted. The distribution of the virtual nodes responding in such large-amplitude limit cycles changes depending on the condition of the plate characterized by its frequency response function $H_{\textrm{st}}(\omega )$. In contrast, when the gain is excessively low (lower-left light blue region of the Fig. 7(a) and (b)), the entire network becomes asymptotically stable, not only degrading the diversity of the network but also resulting in a highly random output as shown in Supplementary Fig. S2(a) online, which further impairs the damage classification performance. Conversely, when the gain is excessively high (upper-right yellow region of the Fig. 7(a) and (b)), all virtual nodes exhibit limit cycles with an amplitude nearly close to 1 as one can see in Supplementary Fig. S2(b) online. This means that the diversity of the node responses is significantly impaired, leading to a deterioration of the classification performance.

Next, the number of the virtual nodes *N* was varied from 10 to 100, while keeping the virtual node bandwidth ($f_\textrm{w} = 20$ Hz) and the center frequency spacing between adjacent virtual nodes (*Δ f = 10* Hz) constant. This consequently changes the total frequency range covered by the whole set of the virtual nodes. The resultant RMSE is plotted in Fig. 7(c), in which the median of the ten trials is indicated by circles while the maximum and minimum values are shown by error bars. The figure shows that the damage classification error rapidly decreases as *N* increases and reaches lower bound when *N* becomes greater than 50 in both damage cases. This suggests a minimum level of the number of nodes for ensuring good damage classification accuracy.

Finally, in order to investigate the influence of the bandwidth overlap between the adjacent virtual nodes, the virtual node bandwidth $f_\textrm{w}$ was varied from 5 Hz to 100 Hz, while keeping the number of virtual nodes (*N = 100*) and the center frequency spacing between adjacent virtual nodes (*Δ f = 10* Hz) constant. The resultant RMSE is shown in Fig. 7(d), from which it is evident that the best value of the bandwidth was 20 Hz in this case. This indicates that there is an appropriate bandwidth for damage classification, and the appropriate bandwidth may depend on the frequency response function of the target structure $H_{\textrm{st}}(\omega )$. When $f_\textrm{w}$ exceeds 20 Hz, the RMSE shows a sudden increase of the variance. A closer look at the responses of the virtual nodes (see **Supplementary Information** online) shows that when the bandwidth $f_\textrm{w}$ is too large, most of the nodes show limit cycles with amplitudes almost close to one or significantly modulated as shown in Supplementary Fig. S3 online, resulting in a reduction in diversity and temporal stationarity. This phenomenon could be explained as follows: when the band overlap with the adjacent nodes increases due to the increase of $f_\textrm{w}$, the coupling between nodes via feedback becomes over-excessive, and the divergence tendency of some nodes become more easily diffused throughout the network, resulting in most nodes showing similar responses. Moreover, an excessively wide bandwidth is likely to cause frequency component mixing that produces beats.

### Comparison with DNN and LSTM

As demonstrated in the preceding sections, the classification performance of the proposed method is attributable to the nonlinear feedback mechanism. More specifically, the instability introduced by the high-gain feedback inherent in the PR is thought to serve as a kind of feature extractor that accentuates changes in the gain and phase characteristics of the target structure.

In this section, to highlight the advantages of this novel feature extraction approach, a comparison is made with a model in which the nonlinear functions and feedback loop have been removed and instead the linear output layer has been replaced with some neural networks that offers higher classification performance than a linear one. While it is expected that using more complex networks would more likely yield classification performance equal to or better than that of the proposed method, this would also increase computational costs. Given that the proposed method is still in the early stages of development, only preliminary comparisons are presented here; specifically, comparison with a classical DNN and LSTM, upon which the advantages of the proposed method will be discussed. A more comprehensive comparison with a variety of topologies of deep neural networks and even with other machine learning algorithms should be addressed in future work.

DNN and LSTM models were implemented in PyTorch. The same actuator and sensor configuration, the same excitation signal (white noise), and the same frequency division and data length were used to ensure the fairness of the comparison. All input data were standardized to have a mean of 0 and a variance of 1 before being fed into the input layer. The input layer contained 100 nodes, matching the number of virtual nodes. The DNN model comprised two fully connected hidden layers with 64 and 36 nodes, respectively, each followed by a ReLU activation function and 20% dropout, and a sigmoid activation function in the output layer. The batch size was set to 32 with a feature dimension of 100, treating each time step as an independent sample. Consequently, the DNN does not explicitly model temporal dependencies and instead learns only the instantaneous amplitude information. In contrast, for the LSTM model, which consisted of a single LSTM layer with 100 units followed by a fully connected output layer, the input was provided as a single long sequence with a batch size of 1 and a feature dimension of 100, allowing the model to directly capture the full temporal structure of the data. Both models were trained using the Adam optimizer with a learning rate of 0.001, a first-moment decay rate of 0.9, and a second-moment decay rate of 0.999. The mean squared error was used as the loss function, and the number of training epochs was fixed at 20. Although extensive hyperparameter optimization was not performed, preliminary experiments with several parameter configurations confirmed that the performance indexes AUC and $E_{\textrm{rms}}$ did not exhibit meaningful variation.

**Fig. 8:**
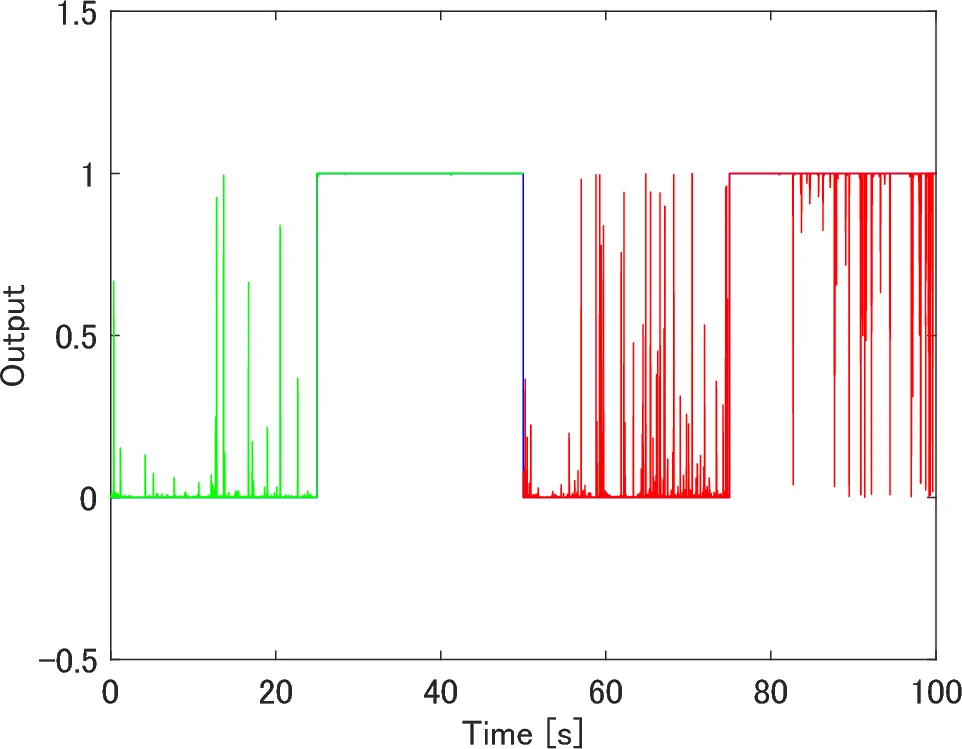
Damage classification results for damage case 1 using DNN. The green and red lines represent the learning results for the training data and validation data, respectively, while the blue line represents the desired output $y_d(t)$. (**a**) Results of the DNN using virtual node signals obtained when the nonlinear function and feedback were disabled as input ($\textrm{AUC} = 1.0000$, $E_\textrm{rms} = 0.0100$).

Figure 8 shows the classification results for damage case 1 obtained using the DNN model. The green and red lines represent the learning results for the training data and validation data, respectively, while the blue line represents the desired output $y_d(t)$. As shown in Fig. 8, the output of the DNN model fluctuates between 0 and 1 indicating that it occasionally fail the classification, resulting in the performance indexes of $\textrm{AUC}=1.0000$ and $E_{\textrm{rms}}=0.0100$. Regarding the training costs, the DNN model contains 8841 trainable weights and requires iterative mini-batch training over multiple epochs to reach convergence. For the LSTM model, the results are not shown because it collapsed to predicting the mean value, i.e., 0.5, regardless of the input, which indicates that it failed to learn meaningful temporal patterns due to insufficient amount of training data.

On the other hand, as shown in Fig. 5(a), although our proposed method demonstrates stability in classification results that is equivalent to or even higher than that of DNNs, it has only 100 trainable weights that can be determined using linear regression, which does not require repeating epochs. This comparison demonstrates the clear advantage of the proposed FVN-PR, that is, a significant reduction in the number of parameters and computational cost. In fact, the number of parameters in this method is approximately 1/90 that of a DNN, which is directly reflected in the inference cost. When using more advanced neural networks, the number of parameters will increase, so this reduction ratio may become even greater. Even more significant is the impact on the training cost. While the proposed method can learn parameters using linear least squares, which does not require iterative computation, DNNs require a number of multiply-accumulate operations proportional to the product of the number of parameters, the number of samples, and the number of epochs. There are even cases, such as with LSTMs, where training is impossible due to insufficient data. In conclusion, the proposed FVN-PR demonstrates a comparable classification performance to that of the conventional machine learning algorithms within the scope of this comparison and offers advantages in terms of significantly lower training and inference costs.

Various lightweight algorithms are being considered to implement damage detection algorithms based on neural networks for edge devices. For example, the research using stochastic configuration networks^44^ has reported a 10 % reduction in parameters through node deletion algorithms; the research incorporating group convolution, depthwise separated convolution, and multi-head self-attention into CNNs has reported a 1/24 reduction in parameters^45^; and the research using knowledge distillation^46^ has reported a 1/40 reduction in parameters relative to the teacher network. The proposed method achieves greater parameter reduction rate than these, suggesting potential advantages as an edge computing technology. Furthermore, as mentioned at the beginning of this section, the proposed FVN-PR can be interpreted as a feature extractor that emphasizes the change in the dynamic characteristics of the structure; from this perspective, rather than competing with neural network technology, it may be possible to use them in combination.

## Experimental verification

To verify the performance of the proposed FVN-PR in practice, evaluation experiments were conducted on two laboratory-scale test specimens: a bolted thin-plate structure and a clamped square-section tube. In the numerical experiments aforementioned, the out-of-plane displacement of the structure was measured at a single point and fed back as a concentrated out-of-plane force at the same point; instead, in the experiments, piezoelectric patches bonded to the structure’s surface were used for both sensing and excitation. Note that this is the functional equivalent of using bending strain instead of out-of-plane displacement, and bending moment instead of out-of-plane force.

### Bolted thin plate structure

#### Two-class classification

**Fig. 9:**
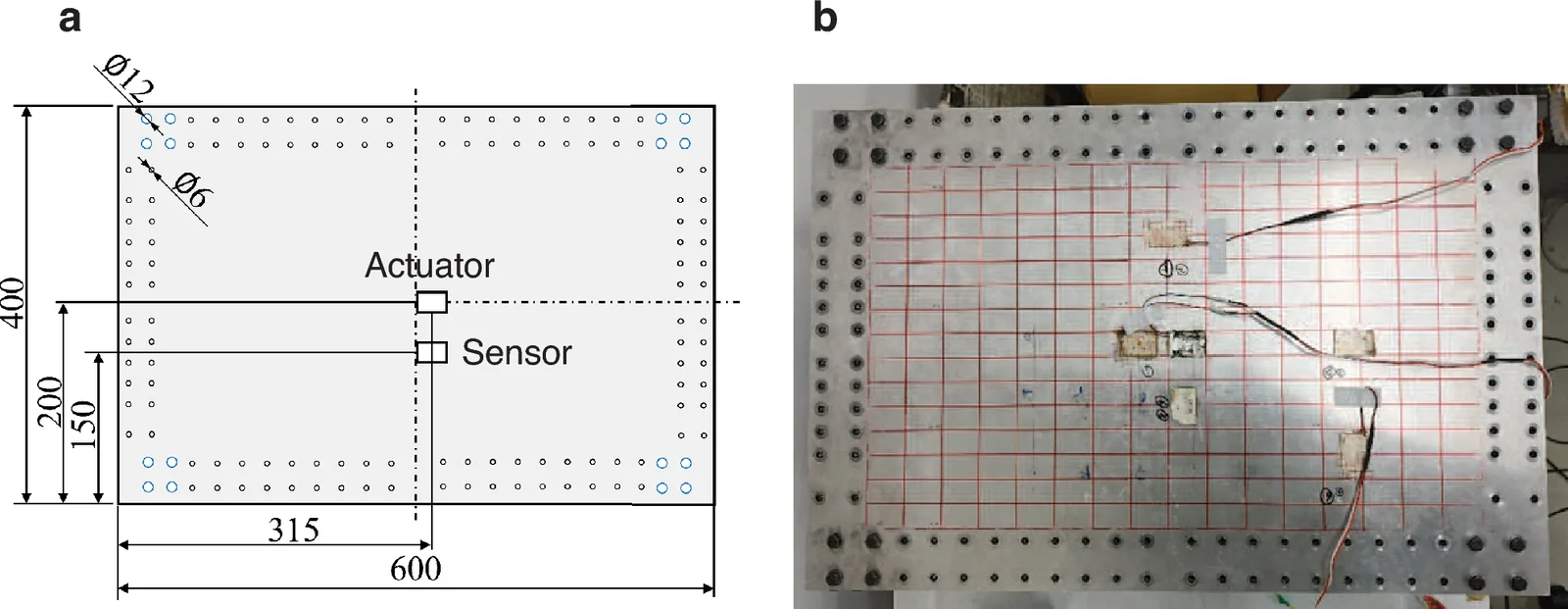
Bolted thin plate specimen. (**a**) Illustration of the thin plate specimen made of aluminum with thickness of 2 mm fixed to a rigid base pillars at its four corners. Two piezoelectric rectangular thin patches of 20 mm *×* 30 mm *×* 0.5 mm were glued on the surface at the indicated locations. For the intact configuration, all the corners were bolted to the pillar with four bolts for each with tightening torque of 8 Nm. For the damaged configuration, one bolt from the lower right corner was removed. (**b**) Photograph of thin plate specimen. The patches not depicted in (**a**) are short-circuited.

In this test, a flat thin aluminum plate shown in Fig. 9 with dimensions of 400 mm in length, 600 mm in width, and 2 mm in thickness was used as a specimen, which was fixed to a rigid base pillars at its four corners with four bolts for each. Two piezoelectric rectangular thin patches of 20 mm *×* 30 mm *×* 0.5 mm are glued on the surface at the locations indicated in Fig. 9(a). For the intact configuration, all the bolts were tightened with the tightening torque of 8 Nm. For the damaged configuration, one bolt from the lower right corner was removed.

The FVN-PR was implemented using a control box equipped with a digital signal processor. Further details are provided in **Supplementary Information** online. Due to the performance limitations of the control box, the number of the nodes was set to 45. White noise was used as input to operate the FVN-PR, and two sets of 25-second node responses were recorded for each health condition; these were used as training and validation data, respectively.

The results presented in Supplementary Fig. S4 online show that the damage classification was achieved at a level comparable to that of the numerical experiments, with a performance index of $\textrm{AUC} = 0.99782$ and $E_\textrm{rms} = 0.0578$. This demonstrates that the proposed method can effectively operate on the actual structure, albeit relatively simple laboratory-scale one.

#### Discussion on performance limits

Little has been discussed regarding the limits of how small damages can be detected. The only known fact is that, when a single bolt was not completely removed but the tightening torque was incrementally reduced, it was not possible to distinguish the condition from the intact state. Therefore, although this is only true for the specific test specimen and implementation, it appears that only relatively large damage, such as the complete detachment of a bolt, can be detectable. However, in general, for damage detection based on changes in dynamic characteristics, the detection limit is typically governed by the frequency band of the vibrations used. Consequently, it is expected that the performance can be improved by shifting the operating frequency band to the higher frequencies or, if possible, extending it up to the ultrasonic range.

#### Multi-class classification

Using the same experimental setup, this time the bolts were removed one by one. Removing one bolt was defined as Case 1, and removing two, three, and four bolts were defined as Case 2, Case 3, and Case 4, respectively. To classify these four damaged cases and the intact state into five classes, a linear output layer with five output neurons was defined and trained. The SoftMax function was applied to the outputs, and the neuron with the highest value was designated as the classification result. Details of the experiment are described in **Supplementary Information** online.

**Fig. 10:**
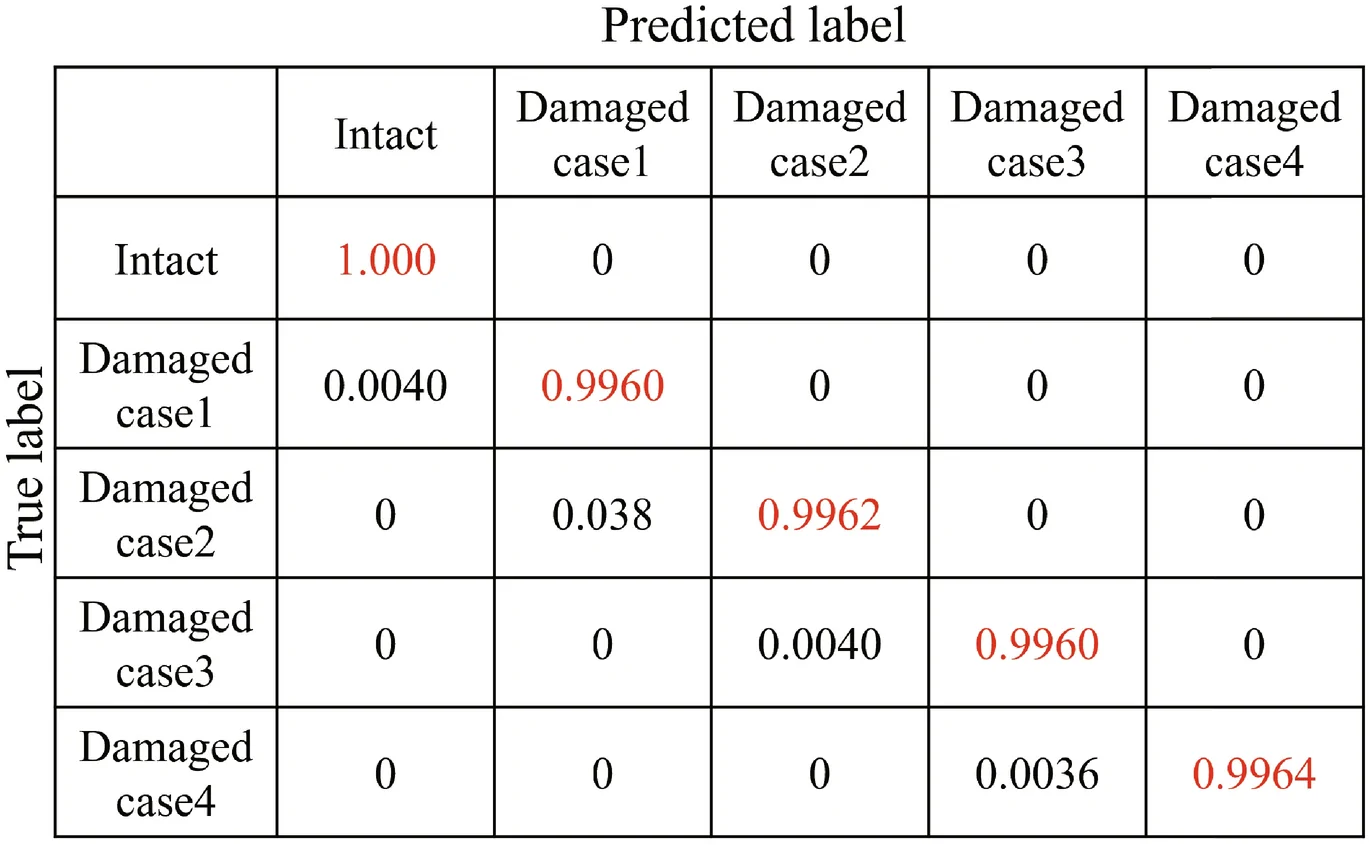
Confusion matrix for multi-class damage classification. The damage states were defined by removing the bolts from the lower right corner one by one. Removing one bolt was defined as Case 1, and removing two, three, and four bolts were defined as Case 2, Case 3, and Case 4, respectively.

The results are summarized in the confusion matrix shown in Fig. 10. All the intact states were correctly classified, and the misclassification rate for the damaged states was less than 0.5 %. Furthermore, the overall accuracy was 99.7 %, demonstrating high classification performance regardless of the damage level.

#### Regression

Returning to the output layer with a single output neuron, training was conducted using the same five healthiness classes as above, assigning incremental values for increasing damage levels. Verification of regression capability for unlearned damage levels was performed in two distinct ways: interpolation and extrapolation. As an interpolation problem, the model was trained by intact and Case 3 damage states, respectively using 0 and 2 as the desired output values. Then it was tested to see if it could produce a consistent value for the unlearned Case 2. Specifically, if the model outputs a value between 0 and 2 for Case 2, it indicates that it has learned an ordinal scale for the degree of damage. On the other hand, as an extrapolation problem, the model was trained by intact and Case 2 damage states, respectively using 0 and 2 as the desired output values, and then tested for its ability to produce a consistent output for the unlearned Case 3. In this case, if the model outputs a value greater than 2 for Case 3, it can be considered a valid ordinal scale for damage severity. Details of the experiment are described in **Supplementary Information** online.

The results are shown in Supplementary Figs. S5 and S6 online. For the interpolation problem, as shown in Supplementary Fig. S5 online, the model produced a value between 0 and 2 (mean value was 0.6064) for the unlearned Case 2, yielding results consistent with the order of damage severity. On the other hand, for the extrapolation problem, the model again produced values between 0 and 2 (mean value was 0.6281) rather than values greater than 2 for the unlearned Case 3 as shown in Supplementary Fig. S6 online. These results suggest that the proposed FVN-PR is effective for interpolative estimation but that quantitative assessment of damage severity is difficult under extrapolative conditions beyond the training range.

### Clamped square-section tube

**Fig. 11:**
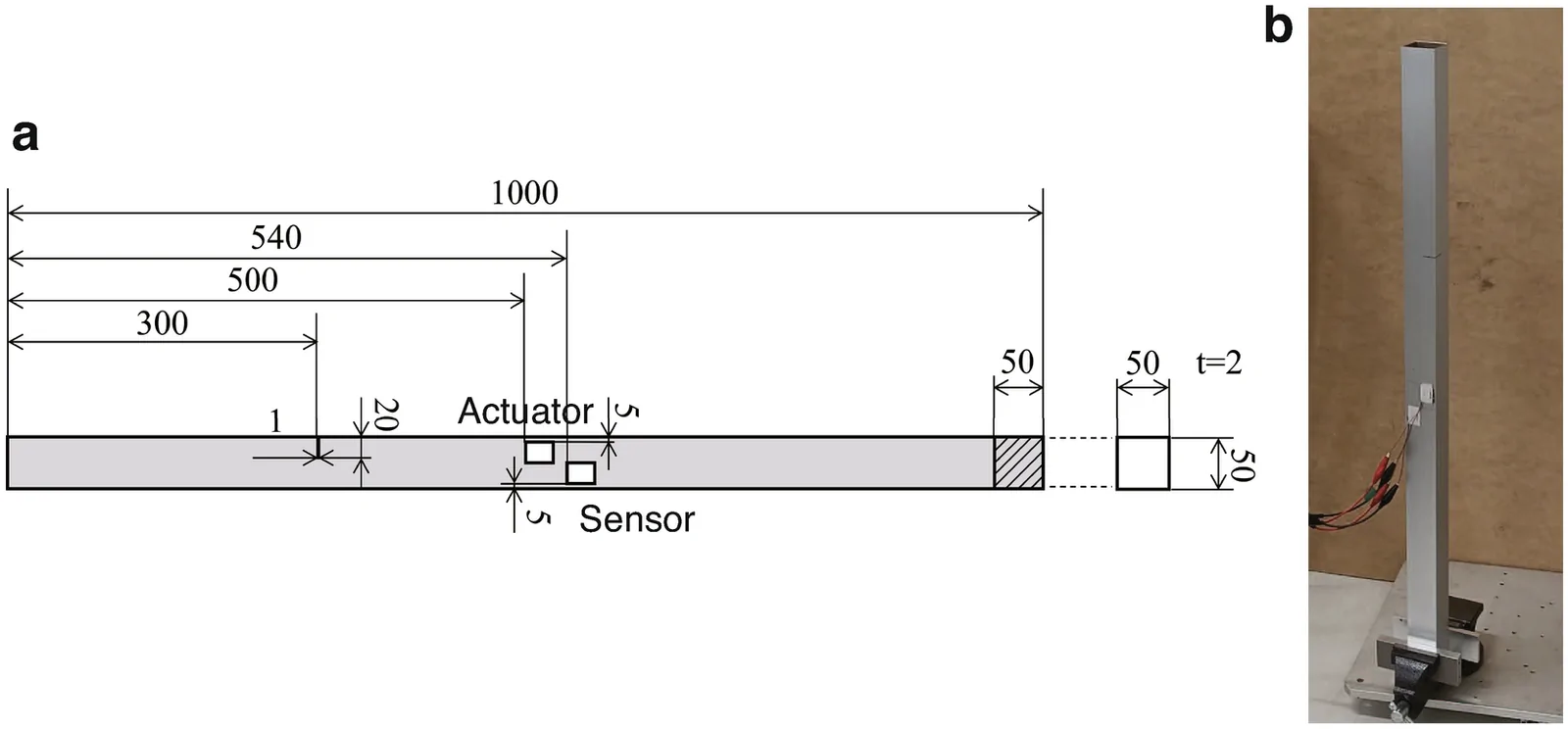
Clamped square-section tube specimen. (**a**) Illustration of the square-section tube specimen made of aluminum with dimensions of 50 mm in height, 50 mm in width, 2 mm in thickness, and 1000 mm in length was clamped at one end with a vice and positioned vertically. Two piezoelectric rectangular thin patches of 20 mm *×* 30 mm *×* 0.5 mm were glued on the surface at the indicated locations. For the intact configuration, a specimen without notch was served. For the damaged configuration, a specimen with a 20 mm long, 1 mm wide notch cut at a position 300 mm from the top end was used. (**b**) Photograph of the specimen.

As another example of a structure different from a flat thin plate, this section employs a thin-walled tubular structure. Figure 11 shows an overview of the square-section tube specimen subjected to the experiment. An aluminum square tube with dimensions of 50 mm in height, 50 mm in width, 2 mm in thickness, and 1000 mm in length was clamped at one end with a vice and positioned vertically. Piezoelectric patches for sensing and actuation were glued at the positions shown in Fig. 11(a). For the intact configuration, a specimen without notch was served. For the damaged condition, a square tube with the same specification was used, having a 20 mm long, 1 mm wide notch cut at a position 300 mm from the top end.

The implementation of the FVN-PR and the experimental details are the same as those in the experiments for the bolted thin plate except for several parameters readjusted. Further details are provided in **Supplementary Information** online. White noise was used as input to operate the FVN-PR, and two sets of 25-second node responses were recorded for each health condition; these were used as training and validation data, respectively.

The results presented in Supplementary Fig. S7 online show that the damage classification was achieved at a level comparable to those of the numerical experiments and experiments for the bolted thin plate, with a performance index of $\textrm{AUC} = 0.99864$ and $E_\textrm{rms} = 0.0506$. This demonstrates that the proposed method can effectively operate on the structure with different shapes.

## Conclusion

In this study, a physical reservoir computing-based framework for structural damage detection, referred to as FVN-PR, in which a single sensor/actuator pair was used along with a combination of the concept of frequency virtual nodes with a nonlinear feedback, was proposed. Numerical experiments conducted on a clamped rectangular aluminum plate successfully learned changes in dynamic characteristics caused by a damage. Furthermore, the method demonstrated a certain level of generalization performance by accurately classifying cases where the damage states of the training and validation data differed. Compared to conventional neural networks such as DNN and LSTM, the proposed method achieved comparable damage classification accuracy with significantly lower training and inference costs.

From the observation of the internal response of the network, it was found that such favorable performance was caused by the diversity of the response amplitudes of the virtual nodes, in particular, by the fact that some of the virtual nodes exhibited large-amplitude limit cycle behavior, and that the distribution of the amplitudes of node responses changed depending on the dynamic properties of the structure. The parameter studies revealed that, in order to create those responses, the network gain and virtual node parameters, such as the number of nodes and bandwidth overlap between nodes, must be appropriately adjusted. In the case of this study, the above situation was achieved when there was an inverse proportional relationship between the common gain *γ* and the feedback gain $G_{\textrm{fb}}$ ($\gamma \times G_{\textrm{fb}} = 0.1$) and the virtual node bandwidth was $f_\textrm{w} = 20$ Hz.

Through experimental validation for two types of laboratory-scale test specimens, it was demonstrated that the proposed FVN-PR can be implemented on real structures and exhibits classification performance comparable to numerical experiments. Furthermore, experiments involving damage levels across multiple stages practically showed its potential for extension to multi-class classification and regression problems (though limited to interpolation problems).

Although it was confirmed that these performances were achieved through the feedback with appropriate gain, non-linear functions, and band-pass filter bank with appropriate overlaps, allowing to realize a system highly sensitive to structural changes, the physical principles behind this is not yet fully understood. In particular, the existence of the feedback incorporating instability is inferred to play a major role, which has not been found in previous SHM approaches; thus it is also the most significant feature that contributes to the novelty of this study. In fact, the conventional SHM, whether passive or active, simply measures the dynamic responses of the target structure and processes them by an external computing process such as neural networks. In contrast, in our approach, the presence of feedback incorporates the structural dynamics as part of the calculation process, significantly reducing reliance on external computational resources. If future research elucidates the underlying physical mechanisms of the proposed method, even more sensitive and real-time damage classification could be achieved at lower computational cost.

There would be other possible variations in the network design. While white noise was used as the input signal in this study, alternative signals, such as sweep waves, continuous impact waves, or vibrations from external environments, could also be considered to sufficiently excites the frequency band of each virtual node. Negative feedback was employed in this study; however, it is hypothesized that positive feedback could also be utilized with appropriate parameter adjustments. Moreover, supposing a general situation where only data on intact structures is available, the expansion to a one-class classification model should be further investigated.

Finally, it is worth mentioning the general applicability and physical implementation of the ability of the FVN-PR demonstrated in this study. In this paper, the damage classification capability of the proposed method was verified only for a simple structures. Nonetheless, the theoretical model was based on modal decomposition, thus in principle, the results obtained in this paper are expected to be applicable not only to plates or thin-walled structures, but also to arbitrary low-damping structures with linear dynamics. The applicability to wider range of structures will be investigated in further study to explore its scope of practical applications.

## Supplementary Information

Supplementary Information.

## Data Availability

The data that support the findings of this study are available from the corresponding author upon reasonable request.
