# Supplementary material for: A physical reservoir involving frequency virtual nodes for structural damage detection
Source: Sci Rep. 2026 Jun 26;16:28227. doi: 10.1038/s41598-026-56840-8 (PMC13558624; doi:10.1038/s41598-026-56840-8)
Supplement: Supplementary file 1 [file 41598_2026_56840_MOESM1_ESM.pdf]

Supplementary information of “A physical reservoir involving frequency virtual nodes for structural damage detection”

Konosuke Takashima[1], Naoko Watanabe[1], Motoaki Hiraga[2], Nanako Miura[2], and Arata Masuda[2,*]

[1]Division of Mechanophysics, Kyoto Institute of Technology, Kyoto, 606-8585, Japan
[2]Faculty of Mechanical Engineering, Kyoto Institute of Technology, Kyoto, 606-8585, Japan
[*]masuda@kit.ac.jp

ABSTRACT

In this document, detailed information of the responses of the virtual nodes is provided for a further understanding of the behavior of the virtual nodes. In addition, detailed information about experimental verification is fully provided.

Responses of virtual nodes

In order to understand how the gains and other parameters affect the damage detection accuracy, the responses of the virtual nodes were closely investigated. As described in the main body of the paper, the default values of the parameters were set as follows: common gain $\gamma = 0.1$, feedback gain $G_{\text{fb}} = 1$, virtual node bandwidth $f_w = 20$ Hz, center frequency spacing between adjacent virtual nodes $\Delta f = 10$ Hz, and number of virtual nodes $N = 100$.

Figures S1(a) and (b) show the absolute values of the temporal responses of all virtual nodes $z_i(t)$ $(i = 1, \ldots, N)$ from $t = 20$ to 20.1, when the parameters were set to their default values. This is the case where the damage detection accuracy is favorable, as displayed by the dark blue region in Figs. 7 (a) and (b). The first thing that can be seen from the figures is that some of virtual nodes have very large amplitudes, while the others have relatively small ones. As can be seen from the comparison of Figs. S1(a) and (b), the amplitude distribution of the virtual nodes varies greatly depending on the state of the structure. Looking more closely, the responses of the virtual nodes with large amplitudes show very regular and periodic responses with amplitudes reaching almost one, even though the entire network is driven by white noise. This means that these nodes exhibit limit-cycle responses, suggesting that destabilization of the network by the feedback and amplitude suppression by the saturating nonlinear function tanh are working simultaneously. Notably, such responses are localized at a few nodes, and their distribution differs between the intact and damaged states of the structure, relying on the frequency response function $H_{\text{st}}(\omega)$. This diversity in amplitude ranges is what provides the stable damage detection performance of the network as seen in Figs. 5 (a) and (b), or in other words, what contributes to the “rich” expressiveness of the proposed physical reservoir.

Next, Fig. S2 (a) shows the responses when the common gain is $\gamma = 10^{-4}$ and the feedback gain is $G_{\text{fb}} = 10^{-2}$, with the structure in the intact state. This set of parameters corresponds to the lower left corner of Figs. 7(a) and (b) belonging to the light blue region. As shown in Fig. S2 (a), the waveform becomes random with very small (in the order of 10^{-3}) amplitude. This is because the system becomes asymptotically stable since the loop gain is extremely small (10^{-6}). Consequently, the node responses lose diversity and regularity, which leads to the low damage detection accuracy. Conversely, Fig. S2 (b) shows the responses when the common gain is $\gamma = 10^2$ and the feedback gain is $G_{\text{fb}} = 10^4$, with the structure in the intact state. This set of parameters corresponds to the upper right corner of Figs. 7(a) and (b) belonging to the yellow region. As shown in Fig. S2 (b), every node responds in a square wave with an amplitude of one. This indicates that all the virtual nodes exhibit limit-cycles with unit amplitude because of the extremely high loop gain (10^6). This time, the node responses are regular but lack any diversity, again losing the characteristics required of the physical reservoir. This is the reason for the worst damage detection accuracy observed in Figs. 7(a) and (b).

Finally, the responses of the virtual nodes for altered bandwidth were investigated. Figure S3 (a) shows the responses when the bandwidth is $f_w = 5$ Hz with the structure in the intact state. As shown in Fig. S3 (a), most nodes respond in a square wave with an amplitude of one except for some nodes responding in smaller amplitude. Meanwhile, Fig. S3 (b) shows the responses when the bandwidth is $f_w = 70$ Hz with the structure in the intact state. In this case, the performance index is the RMSE $E_{rms} = 0.7229$. Figure S3 (b) indicates that, while the low frequency nodes respond in a square wave with an amplitude of one, more than half of the nodes exhibit significant amplitude modulation. This makes the node responses lose diversity and

temporal stationarity, which leads to the low damage detection accuracy.

Experimental verification using bolted thin plate specimen

Specimen and experimental setup for two-class classification

In this test, a flat thin aluminum plate shown in Fig. 9 with dimensions of 400 mm in length, 600 mm in width, and 2 mm in thickness was used as a specimen, which was fixed to a rigid base pillars at its four corners with four bolts for each. Two piezoelectric rectangular thin patches of 20 mm × 30 mm × 0.5 mm are glued on the surface at the locations indicated in Fig. 9(a). For the intact configuration, all the bolts were tightened with the tightening torque of 8 Nm. In the damaged configuration, one bolt from the lower right corner was removed.

The FVN-PR was implemented using a control box (sBOX II; MIS Corporation) equipped with a digital signal processor (OMAP-L137@372MHz; TI) with 16-bit ADC and 14-bit DAC. The measured signal from the piezoelectric sensor was amplified 20-fold before being input to the control box. In the control box, the signal was sampled at 1 kHz, followed by frequency division using a bandpass filter bank, the application of saturation functions, and summation; it was then output via DAC as the feedback signal and amplified 10-fold to drive the piezoelectric actuator. Due to the performance limitations of the control box, the number of the nodes was set to 45. The bandwidth, band interval, total frequency coverage were 10 Hz, 10 Hz, and 20 Hz to 470 Hz, respectively. The feedback gain and the value of γ were set to 6 and 0.01, respectively. White noise was used as input to operate the FVN-PR, and two sets of 25-second node responses were recorded for each health condition; these were used as training and validation data, respectively.

The results are shown in Fig. S4. The green and red lines represent the learning results for the training data and validation data, respectively, while the blue line represents the desired output $y_d(t)$. It is shown that the damage classification was achieved at a level comparable to that of the numerical experiments, with a performance index of AUC = 0.99782 and $E_{\mathrm{rms}} = 0.0578$. This demonstrates that the proposed method can effectively operate on the actual structure, albeit relatively simple laboratory-scale one.

Multi-class classification

Using the same specimen and experimental setup, this time the bolts were removed one by one. Removing one bolt was defined as Case 1, and removing two, three, and four bolts were defined as Case 2, Case 3, and Case 4, respectively. To classify these four damaged cases and the intact state into five classes, a linear output layer with five output neurons was defined and trained. The SoftMax function was applied to the outputs, and the neuron with the highest value was designated as the classification result. For the implementation of the FVN-PR, the number of the nodes was set to 45. The bandwidth, band interval, total frequency coverage were 10 Hz, 10 Hz, and 20 Hz to 470 Hz, respectively. The feedback gain and the value of γ were set to 6 and 0.01, respectively. White noise was used as input to operate the FVN-PR, and two sets of 25-second node responses were recorded for each health condition; these were used as training and validation data, respectively.

The results are summarized in the confusion matrix shown in Fig. 10. All the intact states were correctly classified, and the misclassification rate for the damaged states was less than 0.5 %. Furthermore, the overall accuracy was 99.7 %, demonstrating high classification performance regardless of the damage level.

Regression

Again, the same specimen and experimental setup were used, but the output layer with a single output neuron was adopted. Training was conducted using the same five healthiness classes as above, assigning incremental values for increasing damage levels. Verification of regression capability for unlearned damage levels was performed in two distinct ways: interpolation and extrapolation. As an interpolation problem, the model was trained by intact and Case 3 damage states, respectively using 0 and 2 as the desired output values. Then it was tested to see if it could produce a consistent value for the unlearned Case 2. Specifically, if the model outputs a value between 0 and 2 for Case 2, it indicates that it has learned an ordinal scale for the degree of damage. On the other hand, as an extrapolation problem, the model was trained by intact and Case 2 damage states, respectively using 0 and 2 as the desired output values, and then tested for its ability to produce a consistent output for the unlearned Case 3. In this case, if the model outputs a value greater than 2 for Case 3, it can be considered a valid ordinal scale for damage severity. For the implementation of the FVN-PR, the number of the nodes was set to 45. The bandwidth, band interval, total frequency coverage were 10 Hz, 10 Hz, and 20 Hz to 470 Hz, respectively. The feedback gain and the value of γ were set to 6 and 0.01, respectively. White noise was used as input to operate the FVN-PR, and two sets of 25-second node responses were recorded for each health condition; these were used as training and validation data, respectively.

The results are shown in Figs. S5 and S6. The green and red lines represent the learning results for the training data and validation data, respectively, while the blue line represents the desired output $y_d(t)$. For the interpolation problem, as shown in Fig. S5, the model outputs a value between 0 and 2 (mean value was 0.6064) for the unlearned Case 2, yielding results consistent with the order of damage severity. On the other hand, for the extrapolation problem, the model again produced values

between 0 and 2 (mean value was 0.6281) rather than values greater than 2 for the unlearned Case 3 as shown in Fig. S6. These results suggest that the proposed FVN-PR is effective for interpolative estimation but that quantitative assessment of damage severity is difficult under extrapolative conditions beyond the training range.

Experimental verification using clamped square-section tube

As another example of a structure different from a flat thin plate, this section employs a thin-walled tubular structure. Figure 11 shows an overview of the square-section tube specimen subjected to the experiment. An aluminum square tube with dimensions of 50 mm in height, 50 mm in width, 2 mm in thickness, and 1000 mm in length was clamped at one end with a vice and positioned vertically. Piezoelectric patches for sensing and actuation were glued at the positions shown in the figure. For the damaged condition, a square tube with the same specification was used, having a 20 mm long, 1 mm wide notch cut at a position 300 mm from the top end.

The FVN-PR was implemented using the same control box as the tests for the bolted thin plate. The measured signal from the piezoelectric sensor was amplified 20-fold before being input to the control box. In the control box, the signal was sampled at 1 kHz, followed by frequency division using a bandpass filter bank, the application of saturation functions, and summation; it was then output via DAC as the feedback signal and amplified 10-fold to drive the piezoelectric actuator. Due to the performance limitations of the control box, the number of the nodes was set to 45. The bandwidth, band interval, total frequency coverage were 10 Hz, 10 Hz, and 20 Hz to 470 Hz, respectively. The feedback gain and the value of γ were set to 3 and 0.02, respectively. White noise was used as input to operate the FVN-PR, and two sets of 25-second node responses were recorded for each health condition; these were used as training and validation data, respectively.

The results are shown in Fig. S7. The green and red lines represent the learning results for the training data and validation data, respectively, while the blue line represents the desired output $y_d(t)$. It is shown that the damage classification was achieved at a level comparable to those of the numerical experiments and experiments for the bolted thin plate, with a performance index of AUC $= 0.99864$ and $E_{\mathrm{rms}} = 0.0506$. This demonstrates that the proposed method can effectively operate on the structure with different shapes.

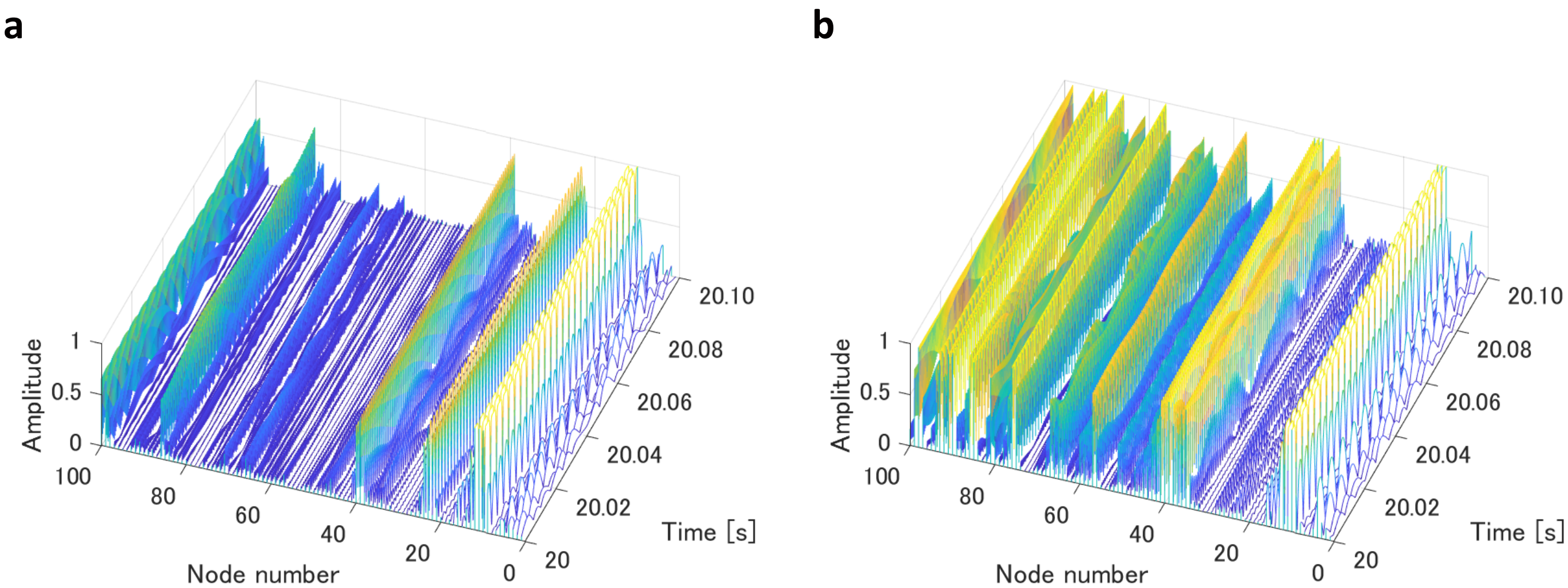

Figure S1. The absolute values of the temporal responses of all virtual nodes $z_i(t)$ $(i = 1, \ldots, N)$ from $t = 20$ to 20.1, when the parameters were set to the default values. (**a**) Responses of all virtual nodes , when the structure is in the intact state. (**b**) Responses of all virtual nodes, when the structure is in the damaged state.

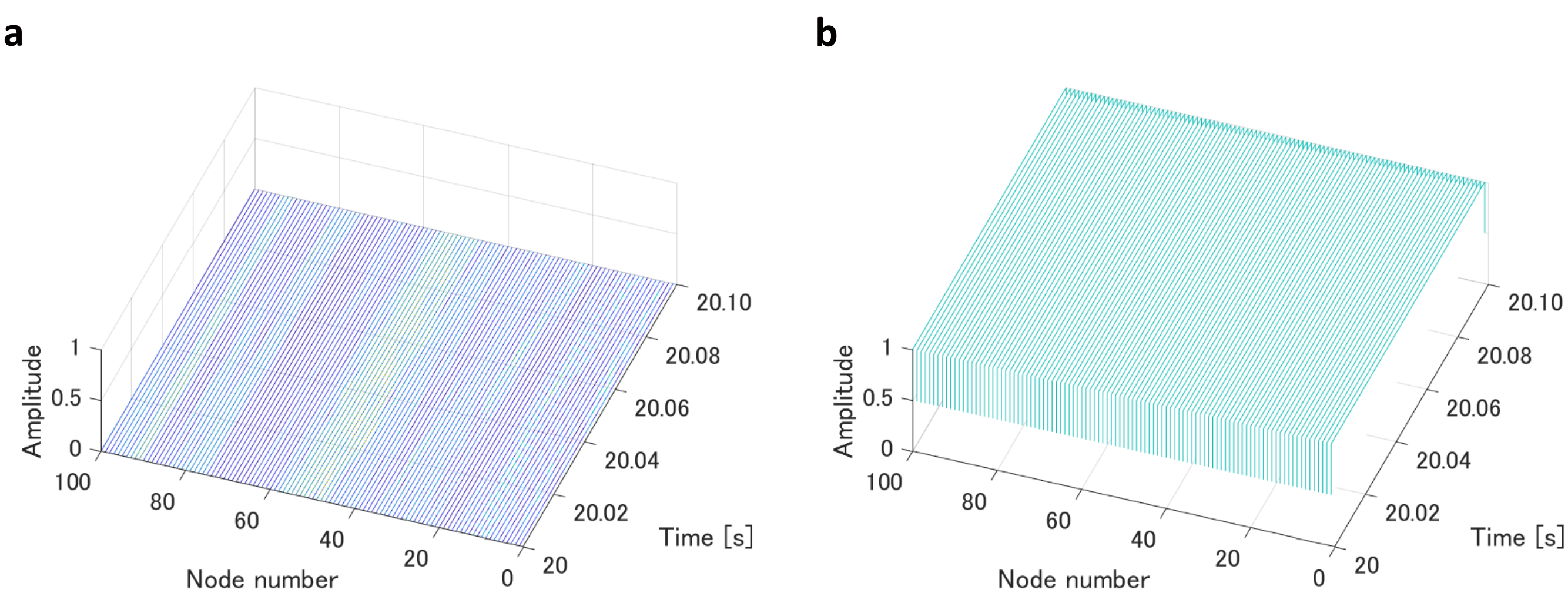

Figure S2. The absolute values of the temporal responses of all virtual nodes $z_i(t)$ $(i = 1, \ldots, N)$ from $t = 20$ to 20.1, when the structure is in the intact state. (**a**) Responses of all virtual nodes when the common gain is $\gamma = 10^{-4}$ and the feedback gain is $G_{\mathrm{fb}} = 10^{-2}$, corresponding to the light blue region in Figs. 7(a) and (b). (**b**) Responses of all virtual nodes when the common gain is $\gamma = 10^{2}$ and the feedback gain is $G_{\mathrm{fb}} = 10^{4}$, corresponding to the yellow region in Figs. 7(a) and (b)

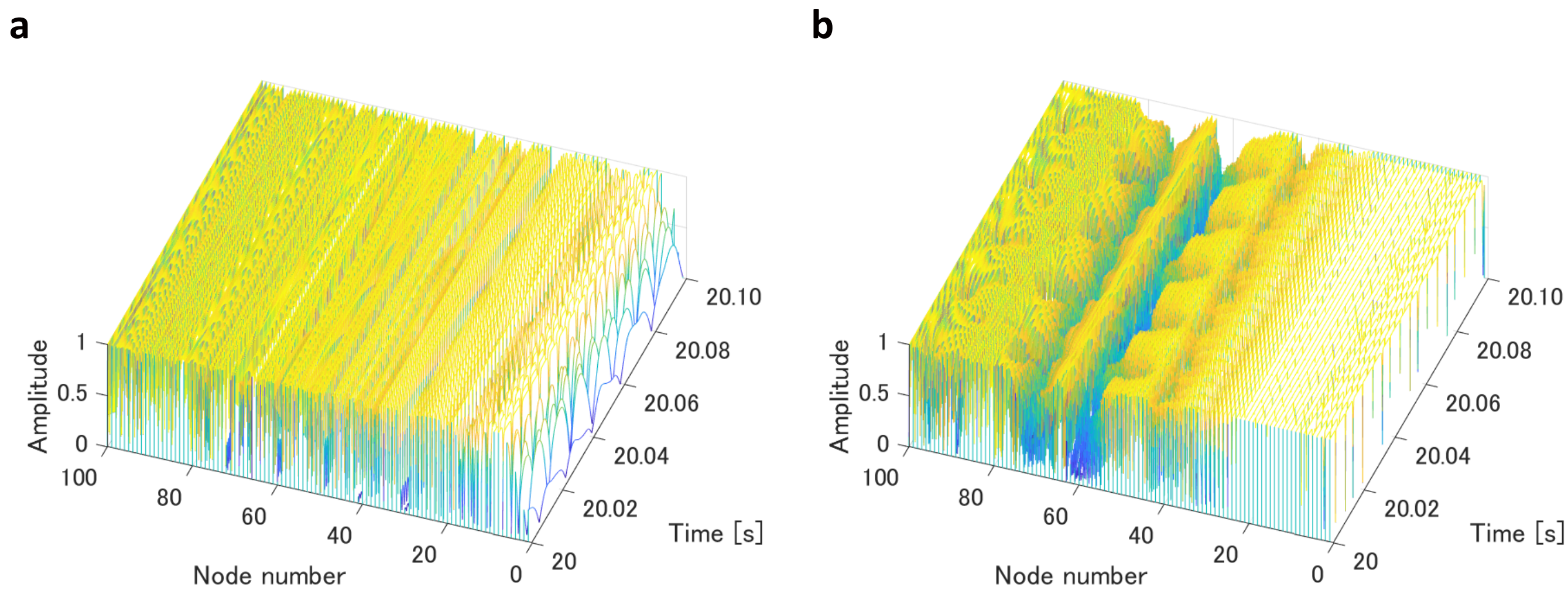

Figure S3. The absolute values of the temporal responses of all virtual nodes $z_i(t)$ $(i = 1, \ldots, N)$ from $t = 20$ to 20.1, when the structure is in the intact state. (**a**) Responses of all virtual nodes when the virtual node bandwidth is $f_w = 5$ Hz. (**b**) Responses of all virtual nodes when the virtual node bandwidth is $f_w = 70$ Hz.

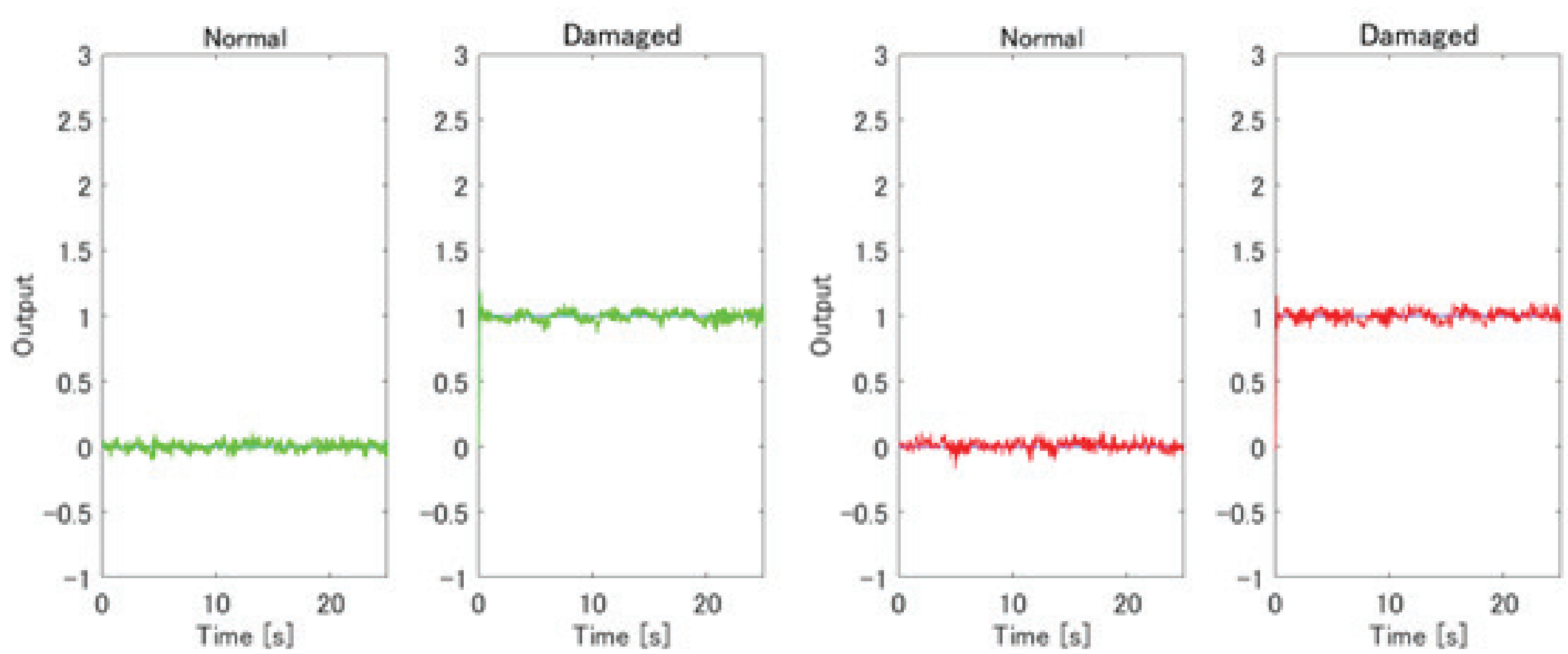

Figure S4. Results of damage classification for the bolted thin plate specimen. The green and red lines represent the learning results for the training data and validation data, respectively, while the blue line represents the desired output $y_d(t)$. The values of the performance indexes were AUC = 0.99934, $E_{\mathrm{rms}} = 0.0554$ for the training data and AUC = 0.99782, $E_{\mathrm{rms}} = 0.0578$ for the validation data.

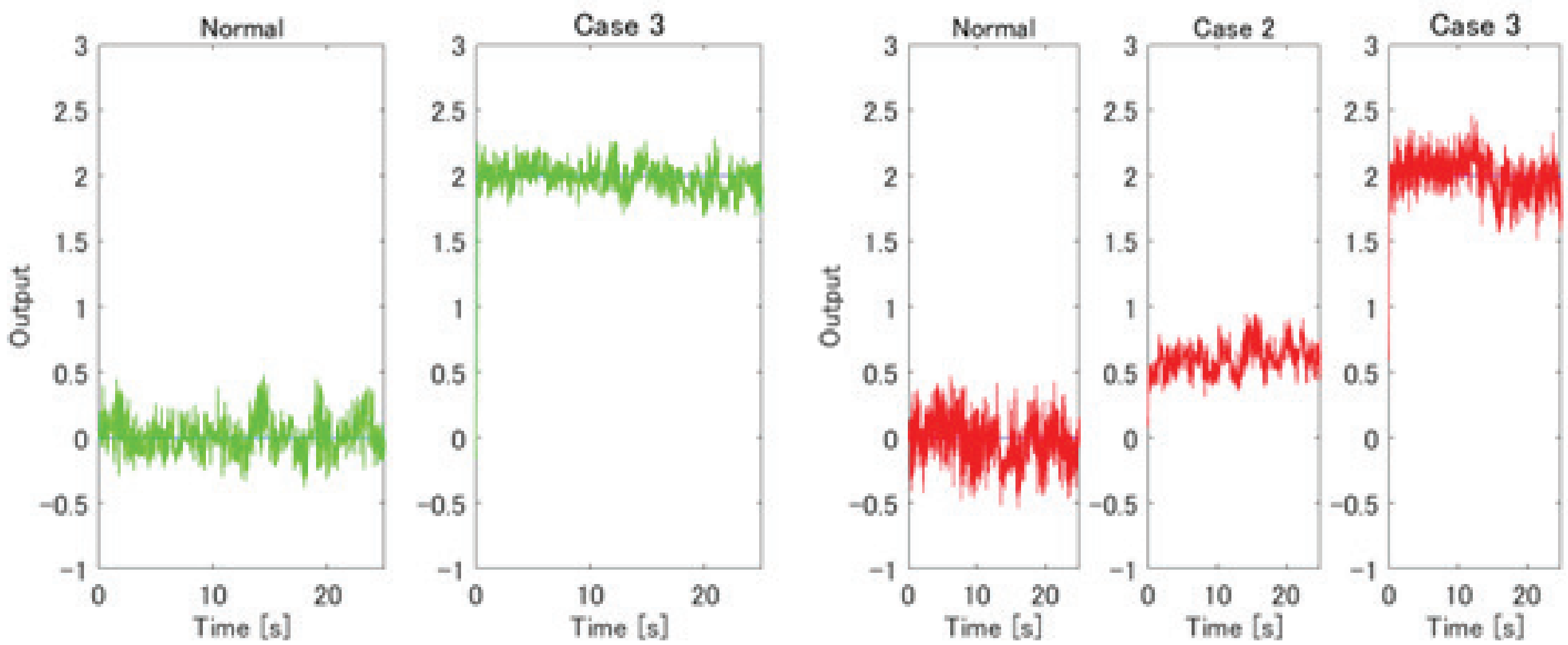

Figure S5. Results for regression (interpolation) of damage severity for the bolted thin plate specimen. The green and red lines represent the learning results for the training data and validation data, respectively, while the blue line represents the desired output $y_d(t)$. The model produced a value between 0 and 2 (mean value was 0.6064) for the unlearned Case 2, which was consistent with the order of damage severity.

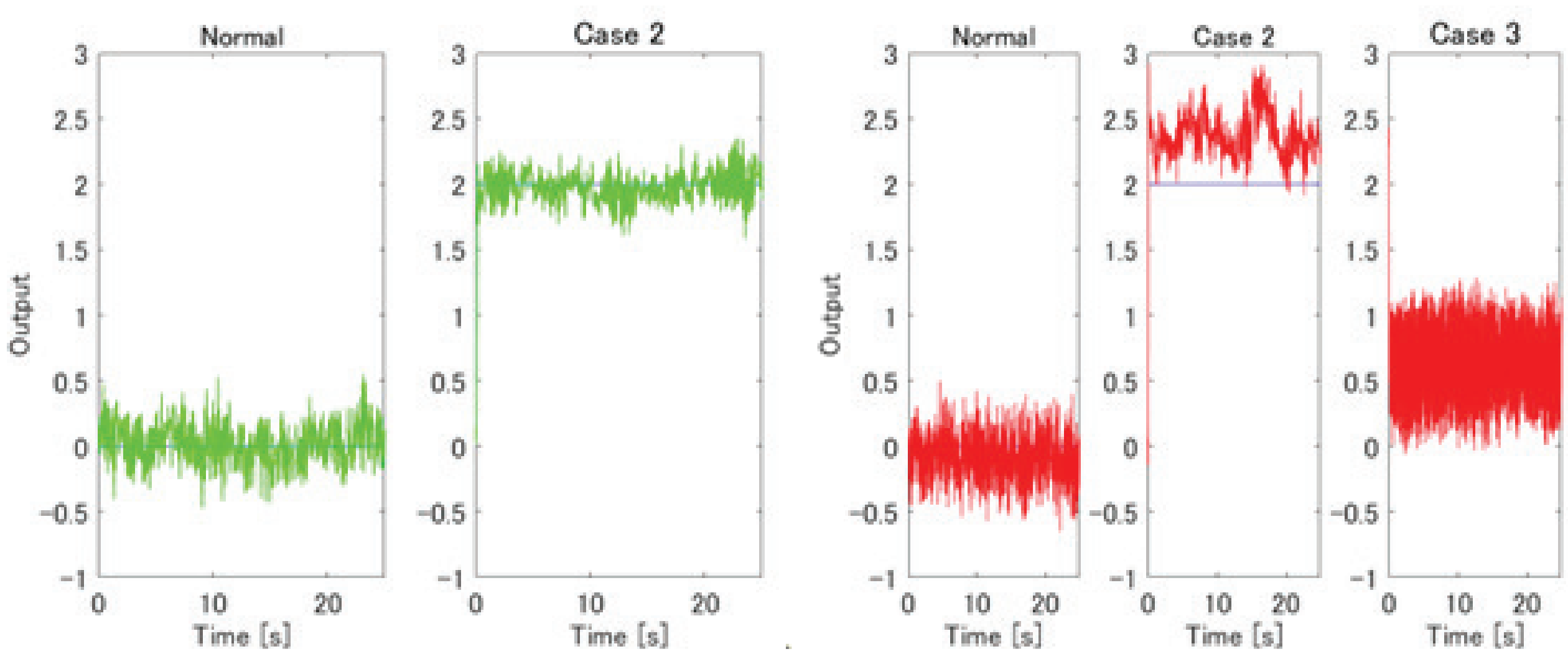

Figure S6. Results for regression (extrapolation) of damage severity for the bolted thin plate specimen. The green and red lines represent the learning results for the training data and validation data, respectively, while the blue line represents the desired output $y_d(t)$. The model produced values between 0 and 2 (mean value was 0.6281) rather than values greater than 2 for the unlearned Case 3, which was inconsistent with the order of damage severity.

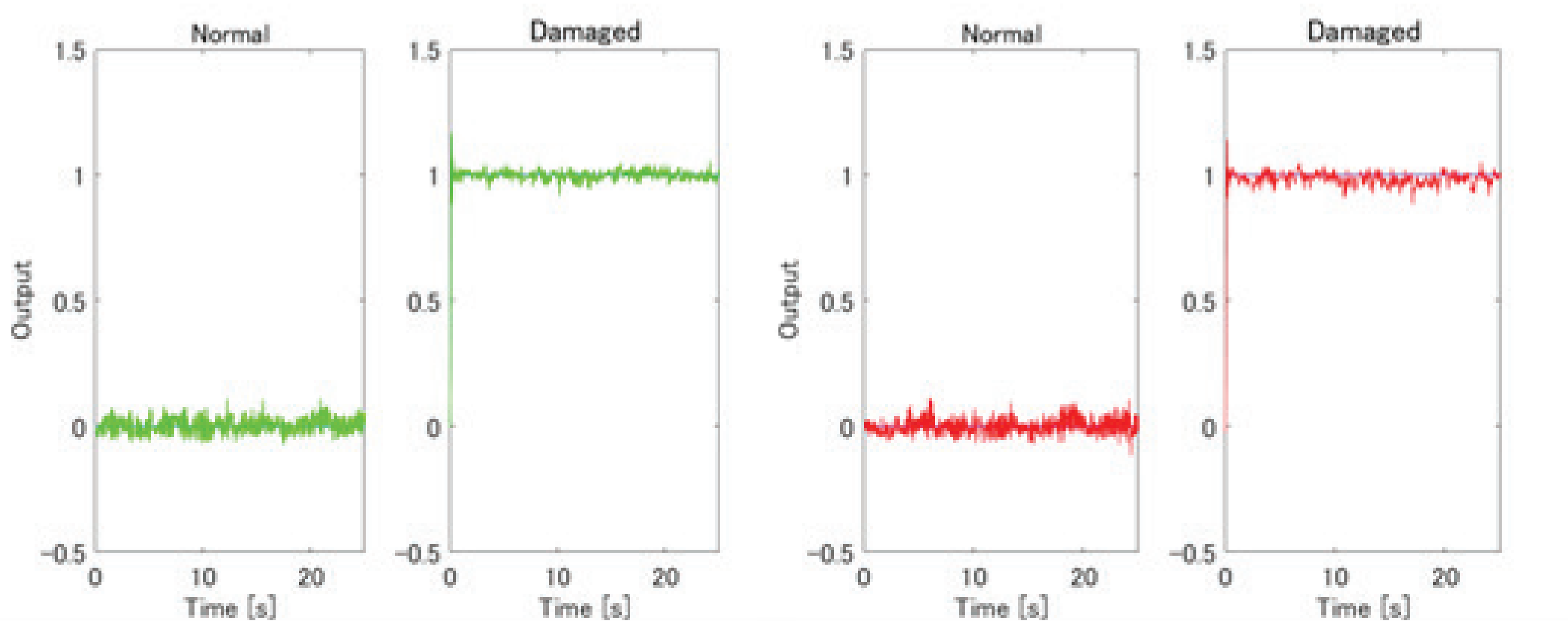

Figure S7. Results of damage classification for the clamped square-section tube specimen. The green and red lines represent the learning results for the training data and validation data, respectively, while the blue line represents the desired output $y_d(t)$. The values of the performance indexes were AUC $= 0.99972$, $E_{\mathrm{rms}} = 0.0435$ for the training data and AUC $= 0.99864$, $E_{\mathrm{rms}} = 0.0506$ for the validation data.
